# Induced Proprioceptor and Low‐Threshold Mechanoreceptor Neurons Derived from Human Pluripotent Stem Cells Exhibit Distinct Functional Mechanosensory Properties

**DOI:** 10.1002/advs.202512413

**Published:** 2025-12-09

**Authors:** Amy J. Hulme, Rocio K. Finol‐Urdaneta, Jeffrey R. McArthur, Nicholas R. Marzano, Simon Maksour, Amarinder Thind, Yang Guo, Dominic Kaul, Marnie Maddock, Oliver Friedrich, Boris Martinac, David J. Adams, Mirella Dottori

**Affiliations:** ^1^ Molecular Horizons University of Wollongong Wollongong NSW 2522 Australia; ^2^ School of Medical Indigenous and Health Sciences University of Wollongong Wollongong NSW 2522 Australia; ^3^ Illawarra Health and Medical Research Institute Wollongong NSW 2522 Australia; ^4^ School of Chemistry and Molecular Biosciences University of Wollongong Wollongong NSW 2522 Australia; ^5^ Victor Chang Cardiac Research Institute Darlinghurst NSW 2010 Australia; ^6^ School of Clinical Medicine St Vincent's Healthcare Clinical Campus University of New South Wales Darlinghurst NSW 2010 Australia; ^7^ Institute of Medical Biotechnology Department of Chemical and Biological Engineering Friedrich‐Alexander University Erlangen‐Nürnberg 91052 Erlangen Germany

**Keywords:** dorsal root ganglia, human pluripotent stem cells, low threshold mechanoreceptor, mechanosensation, NEUROGENIN‐2, PIEZO2, proprioceptor, sensory neuron

## Abstract

Mechanosensory neurons are a specialized class of neurons that detect mechanical stimuli elicited by external or internal body forces. Two major subclasses of mechanosensory neurons reside within the dorsal root ganglia; proprioceptor neurons (PN) that innervate muscle tissue and low threshold mechanoreceptor neurons (LTMR) that innervate skin. To date, the specific cellular neurophysiology of PN and LTMR subclasses are primarily defined by animal models due to the limited availability of human neural tissue. Here an efficient approach is described for generating PN and LTMR from human pluripotent stem cells (hPSC) by inducing co‐expression of NGN2/RUNX3 or NGN2/SHOX2 in hPSC‐derived neural crest, respectively. Molecular and functional mechanosensory profiles are validated in both populations. Of significance, functional interrogation of induced mechanosensory subtypes reveals their distinct responses to mechanical stimuli. Induced proprioceptor neurons produce scaled responses to increasing mechanical stimuli that can sustain repetition and result in action potential firing. In contrast, induced LTMRs desensitize upon repeated mechanical stimuli and display a lower mechanical threshold for action potential firing. Furthermore, both subtypes predominantly rely on PIEZO2 for mechanosensory function. These findings highlight the unique mechanically sensitive profiles and excitability properties that may distinguish human mechanosensory subtypes, distinct from the presence of end‐organs.

## Graphical abstract

This study describes derivation of two distinct mechanosensory subpopulations from human pluripotent stem cells using a transcription factor mediated approach. Of significance, functional interrogation of induced mechanosensory subtypes reveal their unique responses to mechanical stimulation in the absence of end‐organs. These models are highly resourceful to further understand human mechanosensory physiology in healthy and disease contexts.

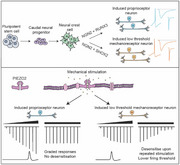

## 1 Introduction

Mechanosensory neurons are specialized to detect and distinguish mechanically evoked stimuli, including stretch, touch, and muscle tension, essential for body awareness of the external and internal environments. Dorsal root ganglia (DRG) mechanosensory neurons are broadly comprised of two major classes: i) proprioceptors (PNs) that detect mechanical signals such as joint position and muscle tension, and ii) low threshold mechanoreceptors (LTMRs) which detect mechanical cues such as touch, hair deflection, and vibration. Further classification of mechanosensory neuron subtypes is multifactorial and includes their innervation of target cells, transcriptional profiles coupled with their functional responsiveness to mechanically induced sensory modalities.^[^ ^1^, ^2^, ^3^, ^4^ ^]^ Loss or dysregulation of mechanosensory neuron functioning results in a range of peripheral neuropathies that can cause chronic pain, ataxia, and/or a loss of touch, bladder, stomach, and sexual sensations. There is a great demand for new strategies to investigate and ameliorate sensory deficits, however, there are major challenges in the identification of potential therapeutic targets. To date, our knowledge of mechanosensory neuron physiology, i.e., electrophysiological signatures and responses to mechanical stimuli, has been determined through rodent studies, due to limited access to human primary DRG tissue. Whilst rodent studies provide valuable insights into mechanosensation, translation of these findings to humans has been challenging due to species‐specific differences in sensory neuronal function.^[^ ^5^, ^6^, ^7^, ^8^ ^]^

Advances in human pluripotent stem cell (hPSC) biology have been a cornerstone for bridging the gap of knowledge in human neuroscience, as differentiation methodologies are becoming increasingly optimised for generating specific neuronal populations. To this end, genetic engineering is growing as a preferable approach for consistently generating homogenous cell types.^[^ ^9^, ^10^ ^]^ The basis of this methodology is to intrinsically regulate the expression of transcription factors that drive cell lineage specification. This strategy has been successfully utilised for generating sensory neurons from hPSC, however, the transcription factors used include NEUROGENIN 2 (NGN2), NEUROGENIN 1 (NGN1), and/or BRN3A, all of which are expressed across multiple DRG sensory subtypes during development.^[^ ^11^, ^12^, ^13^, ^14^, ^15^, ^16^ ^]^ Indeed, findings from our laboratory and others, demonstrate that NGN2‐induced sensory neurons generate a heterogeneous population of DRG subtypes,^[^ ^14^, ^15^, ^16^ ^]^ which confound comprehensive molecular and functional analyses to define specific subtypes in humans. Building on this genetic engineering approach, we hypothesized that combining NGN2 expression with other developmentally relevant transcription factors, such as RUNX3 and SHOX2,^[^ ^17^, ^18^, ^19^, ^20^, ^21^, ^22^ ^]^ would be a strategy to generate specific mechanosensory populations from hPSC‐derived progenitors. In this work, we mimicked sensory neurogenesis via a multistep differentiation protocol, generating hPSC‐derived neural crest cells, followed by the timely induced expression of NGN2 combined with either RUNX3 or SHOX2, to generate induced‐proprioceptors (iPNs) and induced‐LTMRs (iLTMRs), respectively. The iPNs and iLTMRs displayed expression and functional characteristics akin to native proprioceptors and LTMRs, respectively, and responded to different modes of mechanical stimulation (stretch and probe indentation). Of significance, iPNs and iLTMRs both exhibit exquisitely sensitive responses to mechanical stimulation but display differences in their mechanical thresholds and desensitization properties, thereby reflecting their distinct sensory specializations. Furthermore, iPNs and iLTMRs fire action potentials in response to mechanical stimuli and rely on PIEZO2 for mechanosensory function, demonstrating a high level of functional maturity and relevance to human physiology. Overall, our findings describe a unique platform for understanding the fundamental mechanisms that govern distinct responses of human mechanosensory neuronal subtypes to mechanical stimuli that give rise to the perception of touch and tension.

## 2 Results

### 2.1 Expression of NGN2 with RUNX3 or SHOX2 in hPSC‐Derived Neural Crest Cells Induces Molecular Profiles Consistent with PN and LTMR Neurons, Respectively

**Figure 1:**
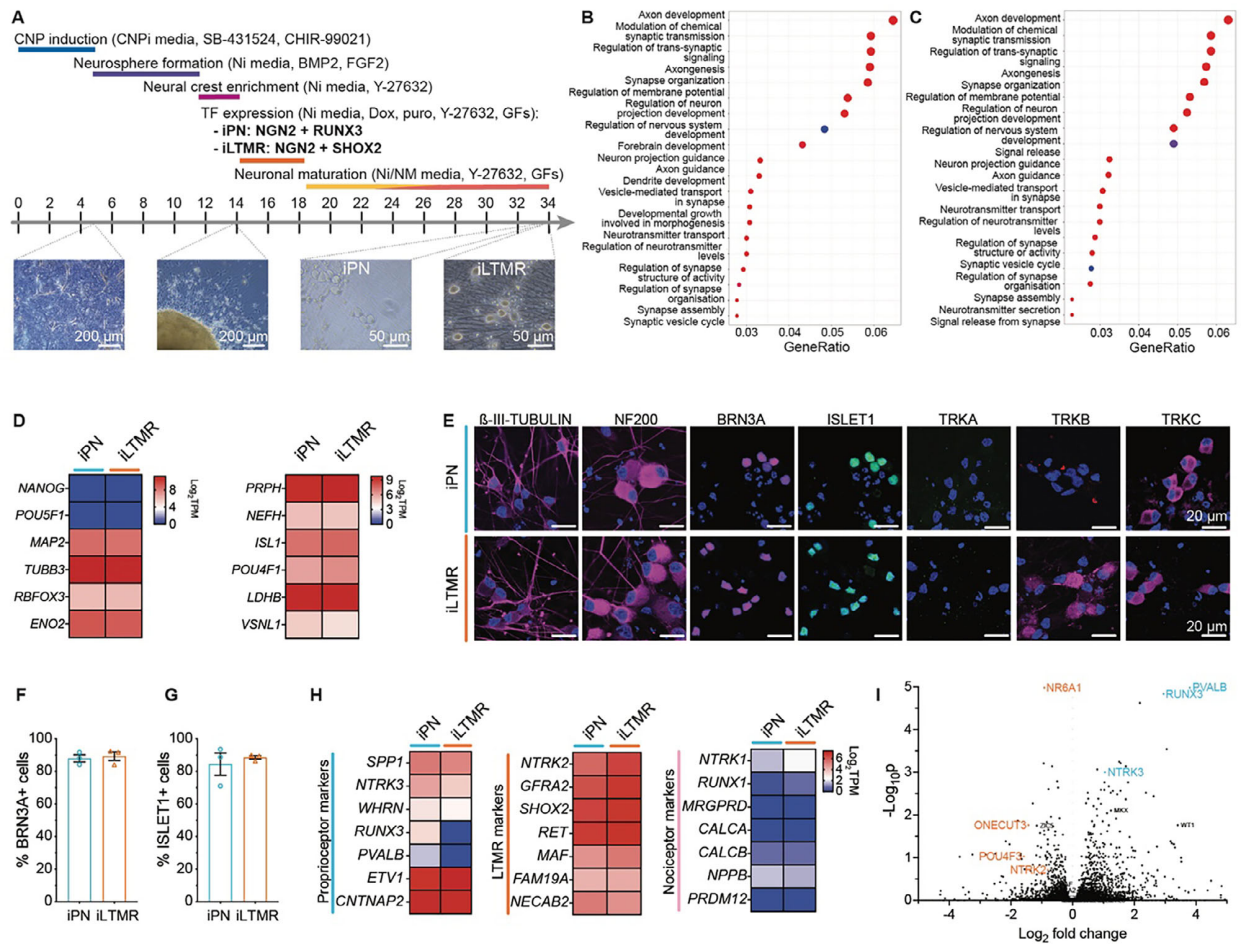
Expression of NGN2‐RUNX3 or NGN2‐SHOX2 in hPSC‐derived neural crest progenitors induces distinct molecular profiles consistent with either proprioceptor and LTMR neurons. A) Schematic of the protocol to derive induced‐proprioceptor neurons (iPN: NGN2+RUNX3) and induced‐LTMR neurons (iLTMR: NGN2+SHOX2). Growth factors (GF) include BDNF, GDNF, NT‐3, and ß‐NGF. Pathways enriched in B) NGN2‐RUNX3 and C) NGN2‐SHOX2 cultures compared to H9 hPSCs. D) Heatmap of key stem cell (*NANOG*, *POU5F1*), neuron (*MAP2*, *TUBB3*, *RBFOX3, ENO2*), and sensory neuron‐specific (*PRPH*, *NEFH*, *ISL1*, *POU4F1*, *LDHB*, *VSNL1*) markers by bulk RNA sequencing (Log_2_TPM) of the iPN and iLTMR cultures, n = 3 biological replicates. E) Representative immunocytochemistry images of the neuronal marker ß‐III‐TUBULIN (*magenta*), the sensory neuronal markers NF200 (*magenta)*, BRN3A (*magenta*), and ISLET1 (*green*), and sensory neuron subtype markers TRKA (*green*), TRKB (*magenta*), and TRKC (*magenta*). BRN3A and ISLET1 are co‐stained images, and overlapping expression of both sensory markers is observed. TRKA and TRKC are co‐stained images, but only TRKC expression is detected. Nuclei are shown in blue. Scale bars = 20 µm. The percentage of iPN and iLTMR cells expressing F) BRN3A and G) ISLET1 compared to the number of nuclei. n = 3 biological replicates, >100 cells counted per biological replicate. H) Heatmap of key proprioceptor, LTMR, and nociceptor markers by bulk RNA sequencing of the iPN and iLTMR cultures, presented as Log_2_TPM, n = 3 biological replicates. I) Differential gene expression plot comparing LTMRs (left) versus PNs (right) as determined by bulk RNA sequencing.

We previously described a robust protocol for generating DRG‐induced sensory neurons from hPSC via induced expression of NGN2 in neural crest progenitors.^[^ ^15^ ^]^ This method produces a heterogenous population of functional DRG sensory neurons that display functional expression of ion channels akin to DRG sensory neurons. Using this approach, hPSCs were initially differentiated into caudal neural progenitors, followed by neural crest cells, and further enriched for migrating neural crest cells (**Figure** **1**A).^[^ ^15^, ^23^ ^]^ To guide the differentiation of these migrating neural crest cells toward populations of mechanosensory neuron subtypes, we transiently induced either *NGN2* and *RUNX3* or *NGN2* and *SHOX2* expression, and the cultures were then further matured to neurons (Figure 1A; Figure S1, Supporting Information). The resulting induced sensory neuronal cultures exhibited molecular profiles consistent with mechanosensory neurons (Figure 1). Enrichment plots comparing the NGN2‐RUNX3 and NGN2‐SHOX2 cultures to H9 hPSCs showed an enrichment of neuron pathways and a high expression of RNA transcripts for the pan‐neuronal markers *MAP2*, *TUBB3* (ß‐III‐TUBULIN), *RBFOX3* (NeuN), and *ENO2* (Neuron‐Specific Enolase) (Figure 1B–D). Evidence of robust DRG sensory neuronal differentiation was supported by a high expression of sensory neuron markers *PRPH* (PERIPHERIN), *NEFH* (Neurofilament heavy chain 200, NF200), *ISL1* (ISLET1), *POU4F1* (BRN3A), and mechanosensory markers *LDBH* (Lactate dehydrogenase B), *VSN11* (Visinin Like 1) (Figure 1D). A high proportion of BRN3A+ (88% and 89%) and ISLET1+ cells (85% and 89%) were present within the NGN2‐RUNX3 and NGN2‐SHOX2 cultures, respectively (Figure 1E–G; Table S1, Supporting Information). Immunostaining further confirmed sensory neuron identity with strong expression of ß‐III‐TUBULIN and NF200 as well as co‐expression of BRN3A and ISLET 1 (Figure 1E).

To profile the NGN2‐RUNX3 and NGN2‐SHOX2‐induced sensory neuron cultures, we examined gene transcripts commonly expressed in PN, LTMR, and nociceptor subtypes. Reflecting PN and LTMR phenotypes, NGN2‐RUNX3 neuronal cultures displayed high expression of TRKC and low levels of TRKA/TRKB, whereas the NGN2‐SHOX2 neuronal cultures showed high expression of both TRKB/TRKC and low TRKA levels (Figure 1E,H). Both NGN2‐RUNX3 and NGN2‐SHOX2 cultures showed comparable expression levels of mechanosensory neuron‐associated genes, including *ETV1*, *CNTNAP2*, *RET*, *NECAB2*, *FAM19A*, and *GFRA2* (Figure 1H), consistent with findings from human DRG single‐cell RNA sequencing studies.^[^ ^8^ ^]^ Despite this overlap, distinct subtype‐specific signatures were evident. Relatively higher expression of classic PN markers was observed in the NGN2‐RUNX3 neuronal cultures, including *SPP1*, *NTRK3* (TRKC), *WHRN* (WHIRLIN), *RUNX3*, and *PVALB* (PARVALBUMIN) (Figure 1H). In contrast, the NGN2‐SHOX2 neuronal cultures had relatively higher expression of LTMR subtype markers such as *NTRK2* (TRKB), *GFRA2, MAF, RET*, *FAM19A*, and *NECAB2*. Further analyses of differentially expressed genes between the two neuronal groups revealed a significant enrichment of proprioceptor markers, RUNX3, PVALB, and NTRK3, within the in NGN2‐RUNX3 cultures, and LTMR markers, ONECUT3 and POU4F3, were enriched in the NGN2‐SHOX2 cultures (Figure 1I). Additionally, minimal expression of genes associated with nociceptors was detected in both the NGN2‐RUNX3 and NGN2‐SHOX2‐induced neurons (Figure 1H). Overall, the transcriptional profiles of the NGN2‐RUNX3 and NGN2‐SHOX2 cultures aligned with PN and LTMR profiles, respectively,^[^ ^8^ ^]^ and thus were subsequently referred to as induced PNs (iPNs) and induced LTMRs (iLTMRs), respectively.

### 2.2 iPNs and iLTMRs Exhibit Distinct Electrophysiological Signatures and Firing Patterns

**Figure 2:**
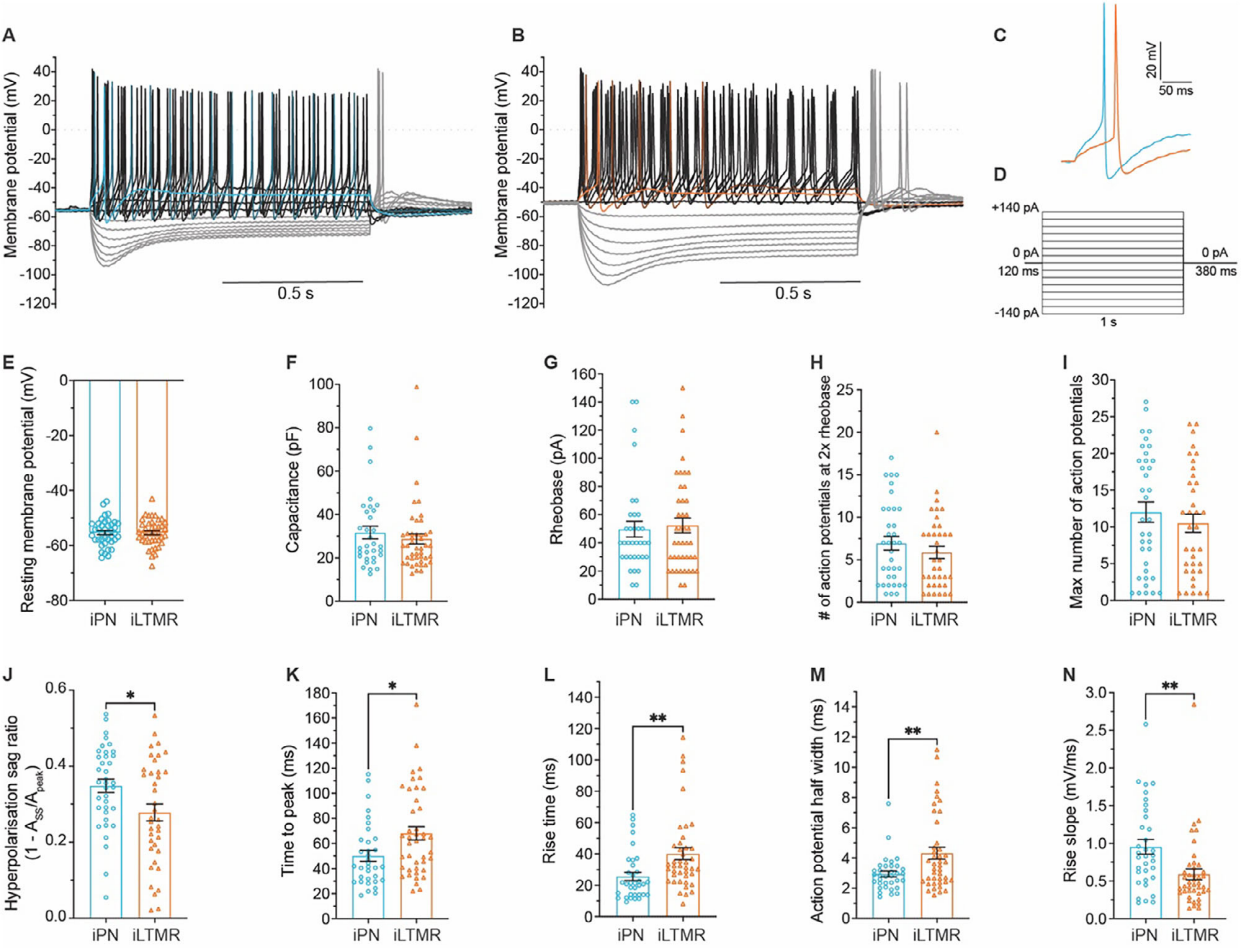
iPN and iLTMR exhibit distinct excitability profiles. Representative current‐clamp recording from A) iPN and B) iLTMR neurons, with C) corresponding action potentials recorded at rheobase. D) Membrane potential responses recorded under current‐clamp conditions elicited by progressive current injections (from −140 to +140 pA, ∆ 10 pA, 1 s, 0.1 Hz). Only every second trace (i.e., Δ 20 pA) is displayed for clarity. Summary of intrinsic excitability parameters for iPN and iLTMR, including E) resting membrane potential, F) membrane capacitance G) rheobase, H) the number of action potentials fired at 2x rheobase, and I) maximum number of action potentials fired. J) Hyperpolarisation sag ratio in response to −150 pA current injection. Action potential properties at rheobase include K) time to peak, L) rise time, M) action potential half‐width, and N) rise slope. Unpaired *t*‐test, ^**^ *p* < 0.01, ^*^ *p* < 0.05. n = 30–45 neurons in total across 7 biological replicates. Data shown represent the mean ± SEM. Full numeric data are provided in Table S2 (Supporting Information).

Neuronal excitability in iPNs and iLTMRs was verified under current‐clamp recording conditions, with incremental current injections (10 pA) (**Figure** **2**, with quantification in Table S2, Supporting Information). iPNs and iLTMRs exhibited robust action potential firing, with brief action potential duration, and increased firing frequency with increasing current stimuli (Figure 2A–D). The induced neuron subtypes shared similar passive membrane properties, with resting membrane potentials of ≈−55 mV, capacitances of ≈30 pF, and rheobases of ≈50 pA (Figure 2E–G). iPNs and iLTMRs fired a similar number of action potentials at two times rheobase and had an average maximum of 12 and 11 action potentials fired, respectively (Figure 2H,I). However, iPNs and iLTMRs displayed distinct active membrane properties, including differences in hyperpolarisation sag ratios upon hyperpolarising current injection (Figure 2J) and action potential shapes (Figure 2J–N). Specifically, at rheobase iLTMR action potentials exhibited significantly longer time to peak, rise time, and action potential half‐width compared to action potentials fired by iPNs (Figure 2K–M). Moreover, iPN action potentials demonstrated faster membrane potential changes in the upstroke (larger rise slope) than action potentials fired by iLTMRs (Figure 2N). Taken together, these distinguishing features suggest differences in the molecular complement underlying the electrically activated excitability features of human‐induced mechanosensory subtypes.

**Figure 3:**
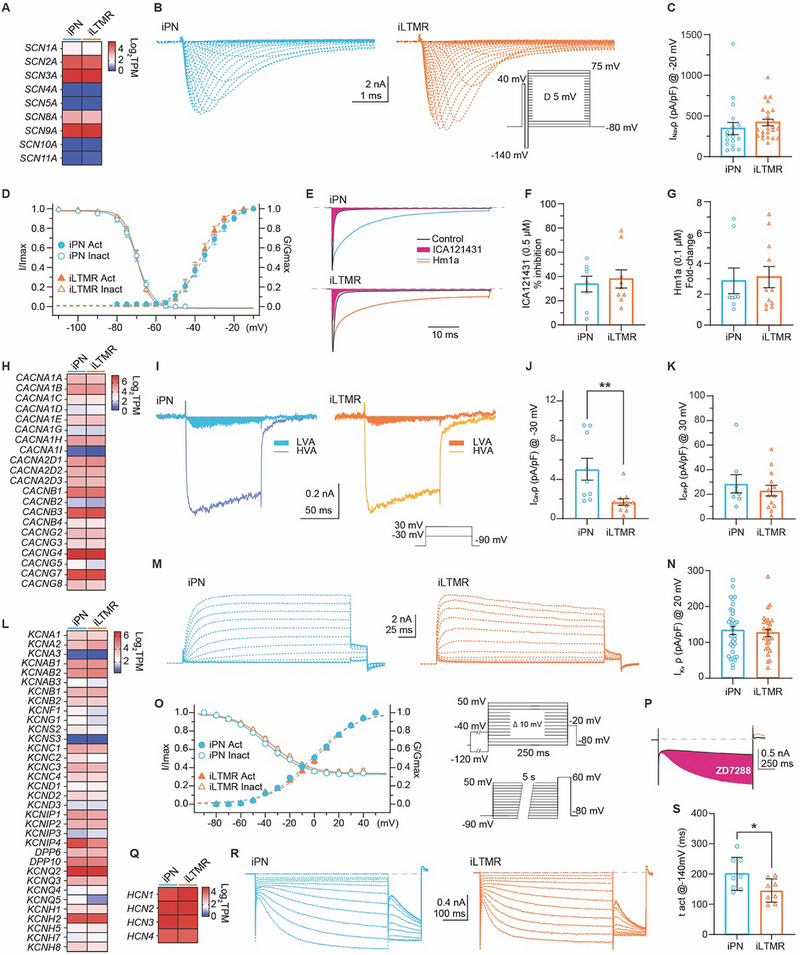
Ionic basis of excitability in iPN and iLTMR. A–G) Depolarization‐activated sodium currents (I_Na_). (A) Expression profiles of transcripts encoding Na_v_ channels in iPNs and iLTMRs. (B) Representative I_Na_ traces recorded from iPNs and iLTMRs. (C) I_Na_ density calculated from peak current at −20 mV. (D) Voltage‐dependence of activation (filled symbols) and steady‐state inactivation (open symbols); insets show stimulation protocols. (E) Representative I_Na_ traces under control conditions (black), following ICA121431 (orange), and Hm1a (blue/gold) application in iPNs and iLTMRs (stimulus: 50 ms, −10 mV, Vh −80 mV, 0.1 Hz). (F) Peak I_Na_ inhibition (%) by ICA121431 (0.5 µm) in iPNs and iLTMRs. (G) I_Na_ enhancement by Hm1a (0.1 µm) in iPNs and iLTMRs. Accumulated charge was estimated by integrating the area under the current (AUC) trace and normalized to the control. H–K) Depolarization‐activated calcium currents (I_Ca_). (H) Expression of calcium channel subunit transcripts in iPNs and iLTMRs. (I–K) LVA Ca_v_‐mediated currents are larger in iPNs compared to iLTMRs. (I) Representative traces showing low voltage‐activated (LVA; filled) and high voltage‐activated (HVA) I_Ca_ in iPN and iLTMRs. LVA (J) and HVA (K) current densities (pA/pF) calculated from peak currents at −30  and 30 mV, respectively. Insets show stimulation protocol. (L‐O) Voltage‐gated potassium channel currents (I_K_) in iPNs and iLTMRs. L) Expression of transcripts encoding K_v_ channels and auxiliary subunits. M) Representative I_K_ traces recorded in iPNs and iLTMRs. N) I_K_ density (pA/pF) calculated from peak current at +20 mV (Vh = −120 mV). O) Voltage dependence of I_Kv_ activation (filled symbols) and steady‐state inactivation (open symbols). The inset shows stimulation protocols. (P‐S) Hyperpolarization‐activated cyclic nucleotide (HCN) channel currents (I_h_) in iPNs and iLTMRs. P) Representative I_h_) traces from iPNs and iLTMRs, recorded in control and following 10 µm ZD7288 application (1 s, −140 mV, Vh = −50 mV, 0.1 Hz). Q) Expression transcripts encoding HCN channels in iPNs and iLTMRs. R) I_h_ activates more slowly in iPNs than in iLTMRs. Families of I_h_ traces in response to 1 s hyperpolarizing steps from −50 to −140 mV (Δ10 mV; Vh −50 mV; 0.1 Hz). S) Activation time constant (τ_act_) from exponential fits to I_h_ at −120 mV (unpaired t‐test). All data are presented as mean ± SEM. Numerical data are provided in Table S3 (Supporting Information).

The overall shape and duration of the action potential result from the complex interplay of various ion channels, including sodium, potassium, and calcium channels. Thus, we analysed voltage‐gated sodium, calcium, and potassium conductance in iPNs and iLTMRs under whole‐cell voltage‐clamp (**Figure** **3**, with quantification in Table S3, Supporting Information). Voltage‐gated sodium channels are crucial for mechanosensation, enabling the transmission of touch and pressure‐related sensory information in neurons. iPNs and iLTMRs exhibited robust voltage‐activated excitatory Na^+^ conductance (> 350 pA/pF, Figure 3B,C), with −36  and −60 mV for half‐activation and half‐inactivation voltages, respectively (Figure 3D; Table S3, Supporting Information). These currents were completely inhibited by 500 nM tetrodotoxin (TTX) (Figure S2, Supporting Information), consistent with the abundant expression of transcripts encoding TTX‐sensitive (TTX‐S) Na_v_ channels and low expression of transcripts (*SCN5A*, *SCN10A*, and *SCN11A*) encoding TTX‐resistant (TTX‐R) Na_v_ channels (Figure 3A). Furthermore, we functionally confirmed the presence of Na_v_1.1 in both induced mechanosensory subtypes, which is consistent with its expression and function in native proprioceptors and LTMRs^[^ ^4^, ^24^ ^]^ (Figure 3E–G).

Crucial for touch and pressure sensation, voltage‐gated calcium (Ca_v_) channels permit Ca^2+^ influx, influencing neurotransmitter release and facilitating signal initiation and transmission in response to mechanical stimuli. iPNs and iLTMRs exhibited abundant expression of Ca_v_ channel transcripts (Figure 3H). Notably, low voltage‐activated (LVA, T‐type) Ca_v_3 channels impact action potential genesis and relay triggered by low‐intensity stimuli. These currents, while small, were notably larger in iPNs compared to iLTMRs during low depolarization (−30 mV) (Figure 3I,J). Based on the transcriptomes, iPN and iLTMR LVA currents correlate with the presence of transcripts of T‐type channels *CACNA1G* (Ca_v_3.1), *CACNA1H* (Ca_v_3.2), and *CACNA1I* (Ca_v_3.3) (Figure 3H). Furthermore, robust high voltage‐activated (HVA) Ca^2+^ currents were evoked during high depolarizations (30 mV, Vh −90 mV, Figure 3K) in both iPNs and iLTMRs (Figure 3I), aligning with the high expression of *CACNA1B* (Ca_v_2.2) and *CACNA1A* (Ca_v_2.1) transcripts (N‐ and P/Q‐type) (Figure 3H).

Voltage‐gated potassium (K_v_) channels regulate neuronal excitability and safeguard cellular homeostasis by influencing the resting membrane potential and membrane repolarization. Potassium currents are mediated by a vast family of membrane proteins actively modulating the characteristics of action potentials, including their shape, duration, and frequency. In iPNs and iLTMRs, abundant expression of multiple K_v_ channels and their modulatory subunits was detected (Figure 3L). Functionally, whole‐cell patch clamp recordings of total K^+^ currents from iPN and iLTMRs revealed substantial outward currents (>125 pA/pF) largely dominated by delayed rectifier K_v_ types (Figure 3M,N). These currents exhibited half‐activation and inactivation voltages of 0  and −36 mV, respectively (Figure 3O; Table S3, Supporting Information).

Hyperpolarization‐activated cyclic nucleotide‐gated (HCN) channels generate hyperpolarizing currents affecting neuronal excitability and processing of mechanical stimuli. All HCN family transcripts were detected in iPNs and iLTMRs (Figure 3Q). Additionally, iPNs and iLTMRs supported robust ZD7288‐sensitive (Figure 3P), hyperpolarization‐activated inward currents (≈18 pA/pF), likely mediated by HCN channels, with half‐activation potentials of ≈−100 mV (Figure 3R; Table S3, Supporting Information). Notably, iPNs displayed significantly slower activation kinetics of HCN currents (at −140 mV) compared to iLTMRs under the same experimental conditions (Figure 3S), providing a plausible basis for the larger sag ratio detected in iPNs during current‐clamp experiments (Figure 2J).

In summary, the absolute current densities of key voltage‐gated ion channels in iPNs and iLTMRs were indistinguishable, with the notable exception of apparently larger LVA Ca_v_ currents, and faster activating HCN currents in iPNs compared to iLTMRs (Figure 3; Table S3, Supporting Information), highlighting subtle distinguishing features between these human mechanosensory neuronal subtypes.

### 2.3 Ion Channel Expression Profiles of iPNs and iLTMRs are Consistent with Mechanosensory Function

**Figure 4:**
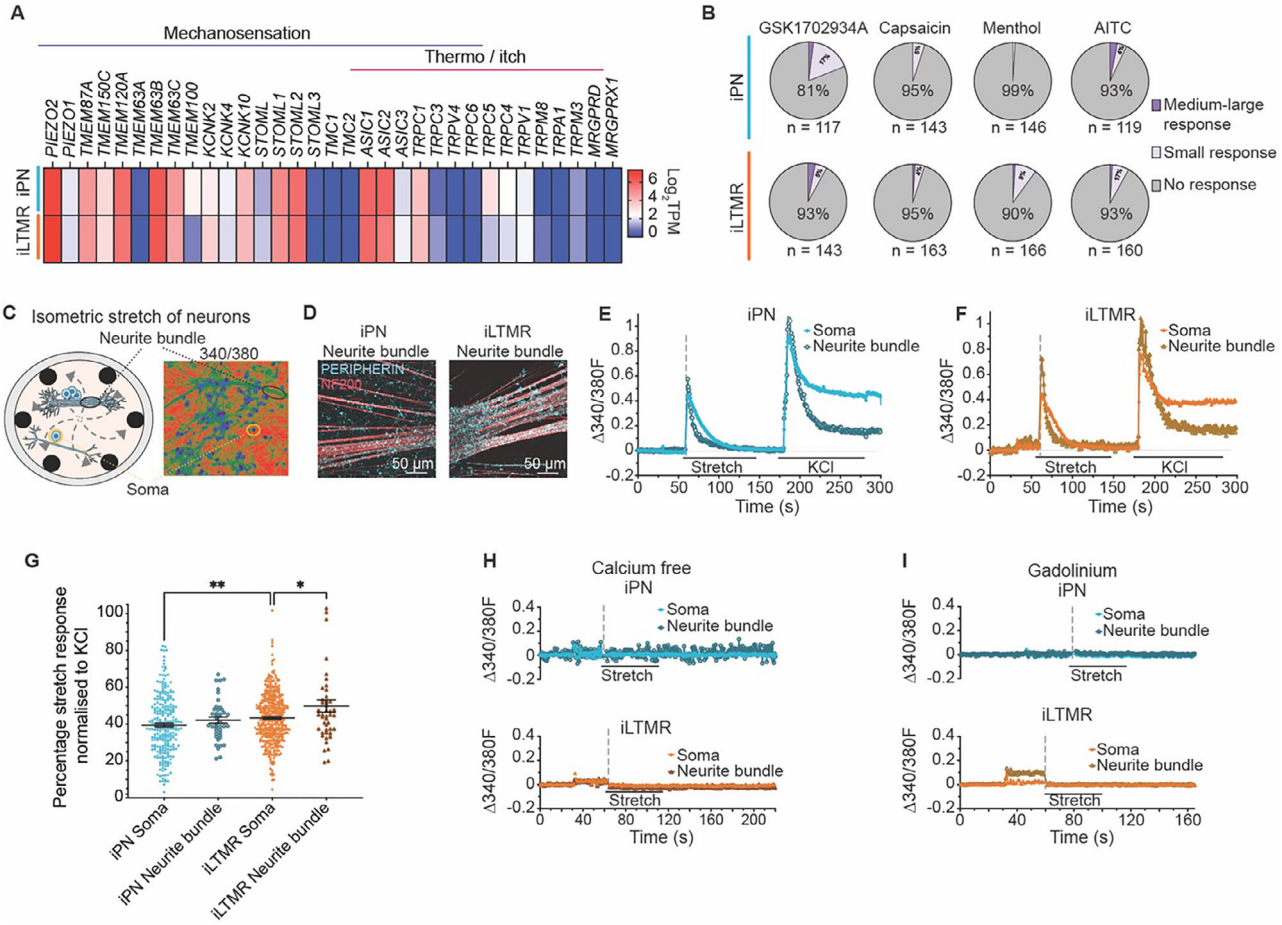
The iPN and iLTMR cultures respond to stretch‐induced mechanical stimulation. A) Heatmap showing expression of gene transcripts associated with mechanosensation and nociception (e.g., temperature and itch) by bulk RNA sequencing (Log_2_ TPM) from iPN and iLTMR cultures (n = 3 biological replicates). B) Proportion of iPNs and iLTMRs responding to mechanical stimuli (stretch) and to nociceptive stimuli (GSK1702934A, capsaicin, menthol, AITC), averaged across n = 3 biological replicates. C) Schematic of iPN and iLTMR somas and neurite bundles cultured on *IsoStretcher* PDMS chambers, measured through Fura‐2 calcium imaging. D) Representative immunocytochemistry images showing PERIPHERIN (*cyan*) and NF200 (*red*) expression in iPN and iLTMR neurite bundles. Scale bar = 50 µm. Representative live cell Fura‐2 calcium imaging traces from an iPN E) and iLTMRF) soma and neurite bundle stretched by 10% via the *IsoStretcher*, followed by 60 mm KCl‐induced depolarisation. Traces represent changes in the 340/380 fluorescence ratio (Δ340/380F) imaged every 0.45 s from baseline of 1 individual neuron and 1 neurite bundle in standard Ca^2+^ buffer solution (CBS). G) Quantification of calcium responses to stretch, normalised to the KCl response. Statistical analysis: one‐way ANOVA with Tukey post‐hoc test, ^**^ *p* < 0.01, ^*^ *p* < 0.05. Data represent mean ± SEM. H) Representative traces of an iPN and iLTMR somas and neurite bundles pre‐incubated in calcium‐free CBS for 20 min prior to 10% stretch stimulation. (n > 80 somas and n > 10 neurite bundles, across n = 3 replicates). I) Representative calcium traces following 5‐min incubation with 300 µm gadolinium prior to 10% stretch in iPN and iLTMR somas and neurite bundles (n > 60 neurons and n > 10 neurite bundles across n = 3 replicates). Numerical data are provided in Table S5 (Supporting Information).

Bulk transcriptomic analyses of iPN and iLTMR cultures revealed abundant expression of ion channels involved in mechanosensation (**Figure** **4**A). *PIEZO2* was highly abundant in both iPN and iLTMR cultures, which is a key mechanosensitive channel expressed in sensory neurons.^[^ ^25^, ^26^ ^]^ Additionally, transcripts of channels that are associated with mechanical sensing were highly expressed in both induced neuronal subtypes, including *TMEM63B*, *TMEM120A* (TACAN), *TMEM87A* (ELKIN1), *ASIC1*, and *ASIC2* (Figure 4A). Notably, iLTMRs showed elevated *KCNK2* (TREK1) expression (Figure 4A; Figure S3, Supporting Information), which functions to dampen responses to mechanical stimulation.^[^ ^27^, ^28^, ^29^ ^]^ Conversely, iPNs exhibited higher levels of *STOML3* and *WHRN* (*Whirlin*) compared to the iLTMRs (Figure S3, Supporting Information), which are known to regulate the sensitivity of mechanically‐gated ion channels and sustained responses to stretch‐evoked stimuli, respectively.^[^ ^30^, ^31^, ^32^ ^]^ Moreover, minimal expression of transcripts encoding ion channels associated with nociception was detected (e.g., *TRPV1*, *TRPA1*, *TRPM8*) (Figure 4A), which was further supported by negligible responses to nociceptive‐like stimuli (GSK1702934A, capsaicin, menthol, AITC) compared to KCl‐induced depolarization (Figure 4B; Figure S5, with quantification in Table S4, Supporting Information). Taken together, the iPNs and iLTMRs express a complement of ion channel transcripts relevant for sensing mechanical cues, indicative of their mechanical sensing function.

### 2.4 iPN and iLTMR Respond to Stretch‐Induced Mechanical Stimuli

A key role of PNs and LTMRs is their capacity to respond to multiple forms of mechanical stimulation, such as stretch and/or indentation. To explore the specific responses of iPNs and iLTMRs to different mechanical cues, we first examined their response to stretch, a low‐threshold mechanical stimulus (Figure 4C). For this, we adapted the *IsoStretcher* and custom‐made Polydimethylsiloxane (PDMS) chambers,^[^ ^33^, ^34^ ^]^ which have been typically used to study ion channel‐mediated mechanosensory transduction in cardiac cells.^[^ ^35^ ^]^ iPN and iLTMR neurons cultured in the PDMS chambers spontaneously clustered together to form “neurite bundles”, which were positive for the sensory markers PERIPHERIN and NF200 (Figure 4C,D). Following maturation, the neuron‐laden chambers were mounted onto the *IsoStretcher* device and were isotropically stretched to 10% (chamber radial increase, ≈20% area increase). The response of both the soma and neurite bundles to stretch was recorded using Ca^2+^ imaging and quantified relative to the KCl response. Accordingly, 100% of the iPN and iLTMR somas and neurite bundles showed robust responses to the stretch‐induced stimuli (Figure 4E,F, with quantification in Table S5, Supporting Information). When normalised to each respective KCl response, the iPN somas and neurite bundles had comparable responses to 10% stretch whereas the iLTMR neurite bundles displayed a significantly larger response to stretch compared to iLTMR somas (Figure 4G). Interestingly, the iLTMR somas had a small but significantly larger response to stretch compared to the iPN somas (Figure 4G). Stretch‐induced fluorescence changes were predominantly supported by extracellular Ca^2+^ influx, as confirmed by minimal changes in Ca^2+^‐free conditions (Figure 4H; Table S5, Supporting Information). Furthermore, inhibition of mechanosensitive channels using gadolinium,^[^ ^36^, ^37^ ^]^ abolished stretch responses in both iPNs and iLTMRs (Figure 4I; Table S5, Supporting Information). Overall, these findings support the functional mechanosensory phenotype of iPNs and iLTMRs.

### 2.5 iPNs and iLTMRs Exhibit Distinct Functional Mechanosensory Properties

**Figure 5:**
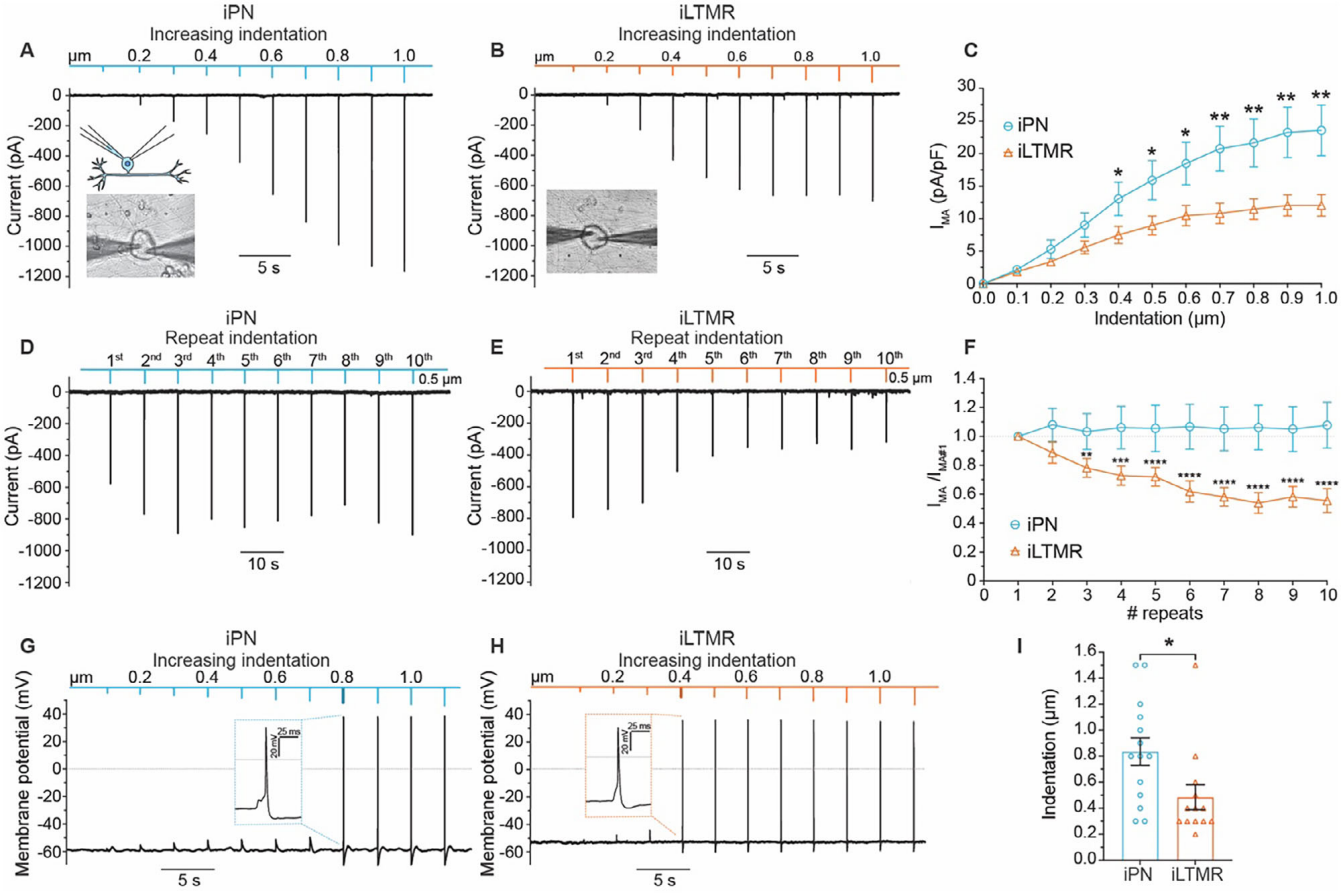
iPN and iLTMR cultures exhibit distinct stimulation‐intensity dependent responses to mechanical stimulation. A,B) Representative whole‐cell voltage‐clamp recordings from an iPN (A) and iLTMR (B) neuron in response to incremental membrane probe indentation (0–1 µm, ∆ 0.1 µm, 1 s duration, 2 s rest between indentations). The inset shows an iPN neuron under whole‐cell patch clamp during probe stimulation. C) Summary of mechanically‐activated (MA) current density (I_MA_) to increased mechanical stimulation by membrane probe indentation steps (0–1 µm, ∆ 0.1 µm) in iPN and iLTMR neurons. Statistical analysis: unpaired *t*‐test, ^**^ *p* < 0.01, ^*^ *p* < 0.05. n = 26–33 neurons across 5–8 biological replicates. D,E) Representative whole‐cell voltage‐clamp recordings from an iPN (D) and iLTMR (E) neuron mechanically stimulated by ten repetitive 0.5 µm indentations (100 ms duration, 5 s rest between stimuli; 0.2 Hz). F) Normalized I_MA_ density (I_MA_/I_MA#1_) in response to repeated 0.5 µm indentations in iPN and iLTMRs. Statistical comparisons to the first indentation: unpaired *t*‐test ^**^ *p* < 0.01, ^***^ *p* < 0.001, ^****^ *p* < 0.0001. n = 15–20 neurons across 4–5 biological replicates. G,H) Whole‐cell current‐clamp recordings from an iPN (G) and iLTMR (H) neuron during progressive membrane probe indentation (0–1 µm, ∆ 0.1 µm, 1 s duration, 2 s rest). Insets show the first action potential triggered by indentation (iPN 0.8 µm; iLTMR 0.4 µm). I) Quantification of mechanical rheobase, the indentation depth (µm) required to elicit the first action potential. Statistical comparison: unpaired *t*‐test, ^*^ *p* < 0.05. n = 13‐14 neurons from 5 biological replicates. Data are presented as mean ± SEM. Numerical data are provided in Tables S6–S8 (Supporting Information).

Given the robust stretch‐evoked responses of iPNs and iLTMRs, we examined whether these neurons could also respond to probe indentation to the soma as an alternative mechanical stimulus (**Figure** **5**, with quantification in Tables S6–S8, Supporting Information). Under voltage‐clamp, iPNs and iLTMRs demonstrated robust mechanically‐activated (MA) whole‐cell currents upon mechanical stimulation to the soma, with stimulation‐intensity dependent increases in the MA current density (denoted as I_MA_) with increasing probe depth (0–1 µm, ∆ 0.1 µm) (Figure 5A–C). In the presence of 300 µm gadolinium, these MA currents were abolished, confirming that the response was due to activation of mechanically sensitive channels (Figure S5, Supporting Information).

iPNs showed heightened sensitivity to depth changes in mechanical indentations, with distinct increases in I_MA_ for each minor (0.1 µm) indentation increment over the entire range of indentations (i.e., 0.1–1.0 um) (Figure 5A,C). In contrast, the average I_MA_ for iLTMRs did not increase substantially with larger indentations of between 0.6 and 1 µm (Figure 5B,C). Furthermore, iPNs responded to mechanical stimulation with a substantially larger I_MA_ than iLTMRs, with approximately double that elicited in the iLTMRs at indentations > 0.4 µm (Figure 5C).

Neuronal desensitization to mechanical stimuli is crucial for regulating the responsiveness of sensory neurons to sustained stimuli. It prevents excessive activation and fatigue of mechanosensitive channels, allowing neurons to adapt to continuous mechanical input while preserving their ability to detect new stimuli. To assess desensitization, we investigated iPN and iLTMR responses to repetitive mechanical stimuli via 10 repeated probe indentations of 0.5 µm (100 ms duration) (Figure 5D,E). While both iPNs and iLTMRs responded to the repeated stimuli, the iPNs maintained similar response amplitudes to repeated mechanical stimulation whereas the iLTMR responses desensitised as evidenced by decreasing responses (Figure 5D,E). iPNs exhibited stable responses with no change in the I_MA_ amplitude with repeated mechanical stimulation compared to the initial mechanical stimulation (defined as I_MA#1_) (Figure 5F). In contrast, iLTMRs demonstrated a significant 22% decrease in the I_MA_/ I_MA#1_ after 3 repeated indentations, which continued to decrease such that after 10 repeats the response was 45% less compared to the initial mechanical stimulation (I_MA#1_) (Figure 5F).

Taken together, iPNs and iLTMRs displayed distinct responses to mechanical stimulation. iPNs elicited discrete MA and increasing responses across the whole range of mechanical stimuli, while iLTMRs’ MA currents plateaued at higher forces and desensitized with repetition.

### 2.6 iPNs and iLTMRs Elicit Action Potentials in Response to Mechanical Stimulation with Distinct Activation Thresholds

A critical role of mechanosensory neurons is the ability to transduce mechanical stimuli into electrical signals, resulting in action potential firing. Thus, we sought to investigate whether iPNs and iLTMRs could recapitulate neuronal firing to mechanical stimuli by membrane probe indentation to the soma (Figure 5G,H). At resting membrane potential with no mechanical stimuli, iPNs and iLTMRs were silent, however, in response to 1 µm mechanical stimuli iPNs and iLTMRs elicited stereotypical single action potentials (Figure S6, Supporting Information). Next, iPNs and iLTMRs were stimulated with progressively larger probe indentations under current‐clamp (i.e., 0.1–1.0 um). Notably, sub‐micrometer membrane indentations resulted in mechanically‐elicited action potentials in both the iPN and iLTMRs somas (Figure 5G,H). Strikingly, iLTMRs were more excitable to mechanical stimuli with a significantly lower indentation threshold compared to iPNs, requiring an average probe indentation of 0.5 µm to elicit an action potential compared to 0.8 µm in iPNs (Figure 5I). These findings demonstrate the ability of iPNs and iLTMRs to transduce mechanical force. The differing levels of sensitivity displayed by iLTMRs and iPNs further highlight their distinguishing functions in mechanosensation.

### 2.7 PIEZO2 is the Major Mechanically‐Activated Sensory Conductance in the Human iPNs and iLTMRs

**Figure 6:**
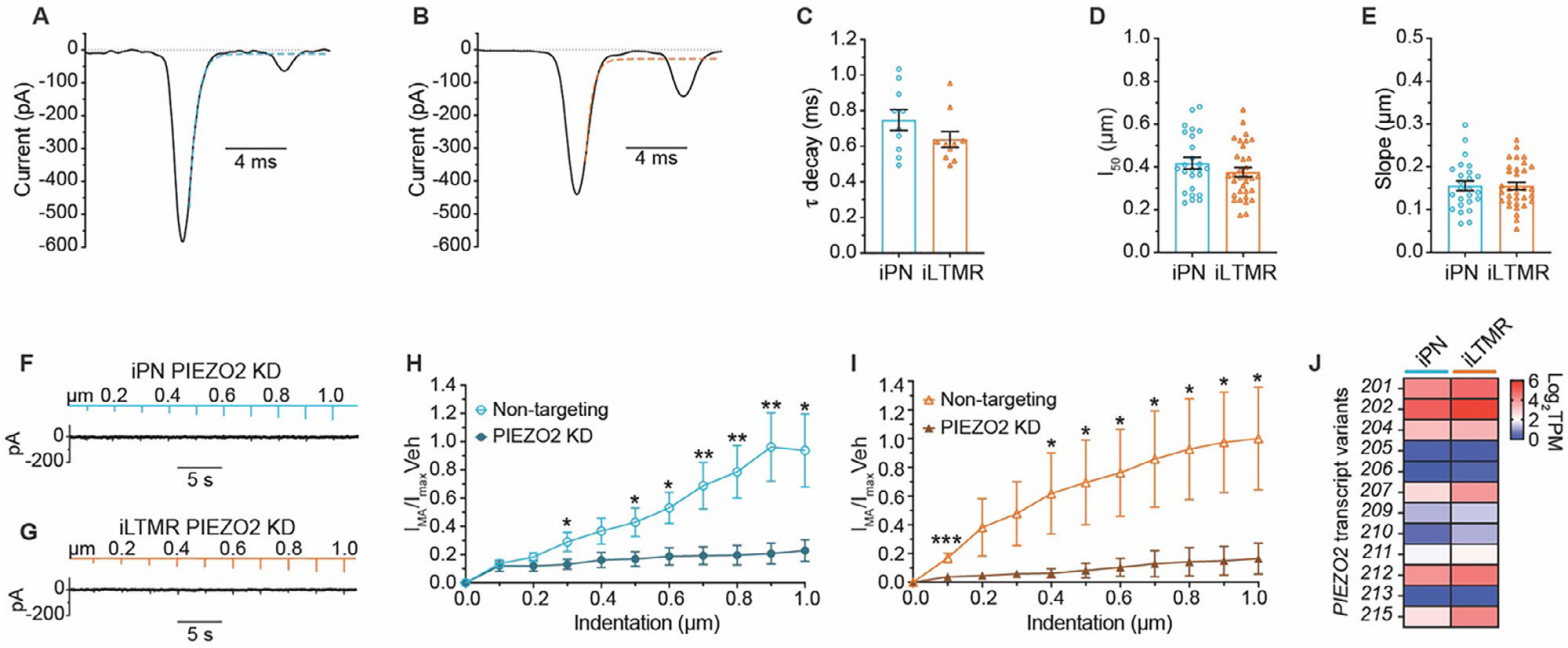
PIEZO2 is a key determinant of mechanosensory modality in iPNs and iLTMRs. A,B) Representative whole‐cell voltage‐clamp recordings from an iPN (A) and iLTMR (B) neuron in response to a 100 ms, 0.5 µm membrane probe indentation. The decay phase of the I_MA_ is fitted with a single exponential function (dotted line). C) Average current decay time constant (τ) of iPNs and iLTMRs, calculated from the exponential fits of MA currents (n = 10 neurons from 3 biological replicates). D,E) Quantification of mechanical activation properties derived from Boltzmann fits: (D) probe indentation depth required to elicit 50% of the maximal current (I_50_) and (E) slope of the activation curve for iPNs and iLTMRs (n = 25–33 neurons from n = 5–8 biological replicates). F,G) Representative traces from an iPN (F) and iLTMR (G) transfected with PIEZO2 siRNA, in response to incremental membrane probe indentation (0–1 µm, ∆ 0.1 µm). H,I) Normalized MA current density in iPNs (H) and iLTMRs (I) transfected with non‐targeting or PIEZO2 siRNA across membrane indentation steps (0–1 µm, ∆ 0.1 µm). Data are normalised to the mean current response at 1 µm in the non‐targeting siRNA condition. Statistical analysis: unpaired *t*‐test, ^**^ *p* < 0.01, ^*^ *p* < 0.05. n = 15–17 neurons from 3 independent siRNA transfections. J) Heatmap showing *PIEZO2* splice variant expression (Log_2_ TPM)in iPN and iLTMR cultures (n = 3 biological replicates). Data are presented as mean ± SEM. Numerical data are provided in Table S9 (Supporting Information).

Mechanically sensitive DRG neurons can be distinguished based on the kinetics of their MA currents, which are classified as either rapidly‐adapting (< 10 ms), intermediately‐adapting (between 10 and 30 ms), and slowly‐adapting (> 30 ms) current decays. These kinetics are determined by specific mechanosensitive channels and their modulators.^[^ ^2^, ^3^, ^31^, ^38^ ^]^ PNs and LTMRs typically display MA currents that decay rapidly, while certain nociceptor subtypes are either mechanically insensitive or exhibit intermediately‐ or slowly‐adapting MA current decays.^[^ ^2^, ^3^, ^38^ ^]^ We applied an exponential fit of the inactivation kinetics in iPNs and iLTMRs (**Figure** **6**A,B, with quantification in Table S8, Supporting Information) and observed that their MA currents decayed rapidly with an average time constant (τ) of 0.75  and 0.64 ms, respectively (Figure 6C). To compare the activation kinetics for both induced mechanosensory subtypes, the current‐displacement relationship was established by normalising the I_MA_ to the observed maximal response (denoted as I_max_) and fit to a Boltzmann equation (Figure S7, Supporting Information). The activation kinetics of iPN and iLTMR I_MA_ were similar, requiring ≈0.4 µm indentations to achieve half‐maximal activation (I_50_) (Figure 6D). Both induced subtypes also displayed comparable mechanosensitivities with slopes of 0.16 (Figure 6E), suggesting that the same channel may be a key mediator of their mechanosensory function.

Given the fast MA current kinetics observed in iPNs and iLTMRs, we next examined the contribution of the PIEZO channels in mediating the iPN and iLTMR mechanosensory responses. *PIEZO2* knockdown significantly reduced iPN and iLTMR MA responses to increasing membrane displacements by up to 75% and 80%, respectively (Figure 6F–I; Table S9, Supporting Information), highlighting the role of PIEZO2 in mediating iPN and iLTMR responses to mechanical stimulation. Furthermore, siRNA knockdown of *PIEZO1*, which is involved in the detection and transduction of mechanical itch in sensory neurons,^[^ ^39^ ^]^ yielded I_MA_ responses indistinguishable from non‐targeting siRNA controls in both subtypes (Figure S8, Supporting Information), indicating that PIEZO1 does not contribute to MA currents in iPNs and iLTMRs. This data suggests the potential involvement of MA channels other than PIEZO1 in iPNs and iLTMRs. Given that most of the MA current was mediated by PIEZO2, we investigated the expression of PIEZO2 splice variants between iPN and iLTMR subtypes. Notably, iPNs and iLTMRs expressed multiple *PIEZO2* splice forms, with differences in the proportions of splice variants (Figure 6J), consistent with the presence of multiple PIEZO2 splice variants within human sensory neurons.^[^ ^40^ ^]^ Taken together, PIEZO2 is the primary mechanosensitive ion channel mediating stimulation‐intensity dependent MA currents in iPNs and iLTMRs, consistent with in vivo PNs and LTMRs.

## 3 Discussion

Mechanosensation is vital for touch, spatial positioning, and internal organ sensations, enabling our body to be aware and interact with our internal and external environments. Specialised mechanosensory neurons detect and discriminate a plethora of internal and external signals, however, the understanding of mechanosensory physiology has predominantly relied on rodent studies and more recently, on cadaver‐explanted, axotomized DRG neurons. In this study, we generated cultures of iPN and iLTMR neurons from hPSCs and profiled their distinct molecular and functional features, which can facilitate the investigation of intrinsic cellular mechanisms within human sensory neuron mechanosensation and how this can be dysregulated in disease.

The multi‐step differentiation protocol described in this study resulted in the generation of specific subpopulations of mechanosensory neurons that were highly sensitive to mechanical stimuli. A key finding is that both iPNs and iLTMRs had exclusively rapidly adapting MA current decays, mediated by PIEZO2, and did not respond to nociceptive stimuli. Furthermore, iPNs and iLTMRs had large MA current responses to < 1 µm indentations and defined responses to submicron mechanical stimulation, which has not been previously characterised in hPSC‐derived neurons. Our data is in contrast to previous differentiation protocols that have induced the expression of broad sensory neuron transcription factors (e.g., NGN1, NGN2, and/or BRN3A), generating heterogeneous populations of PNs, LTMRs, and nociceptors.^[^ ^13^, ^14^, ^15^, ^16^ ^]^ Of these, induced expression of NGN1 in hPSC‐derived neural crest cells results in the generation of neurons with rapidly‐adapting, intermediately‐adapting, and slowly‐adapting MA current decays indicative of the presence of both induced‐LTMRs and induced mechanically‐sensitive nociceptors.^[^ ^13^ ^]^ Furthermore, NGN2‐alone or in combination with BRN3A in hPSC‐derived neural crest cells generates mechanosensitive neurons capable of responding to nociceptive stimuli depending on the length of induced expression.^[^ ^11^ ^]^ Differences in the starting cell type (progenitor vs hPSC vs fibroblast), length of induced expression, and combination of small molecules, growth factors, and transcription factors can dramatically alter the fate of the neurons.^[^ ^9^ ^]^ Importantly, our findings demonstrate the significance of inducing fate‐specifying transcription factors (e.g., NGN2/RUNX3 or NGN2/SHOX2) during key developmental stages (such as neural crest) to drive neuronal differentiation to specific sensory subpopulations.

A limitation of our study was the inability to determine the exact subtype of PN or LTMR the cultures best fit. Typically, mammalian PNs and LTMRs are further sub‐grouped based on the (1) myelination and conduction velocity, (2) location within the body, (3) end organs that the neurons innervate and associate with, (4) distinct physiological characteristics, and (5) the gene expression profile.^[^ ^1^, ^28^, ^41^ ^]^ However, within the context of our system, the comparisons of the iPN and iLTMRs to primary mechanosensory subgroups were instead based on the expression and functional characteristics of the iPNs and iLTMRs. Despite the absence of end organs, iPNs and iLTMRs revealed distinct expression and functional profiles consistent with the generation of neurons with discrete mechanosensory specializations. iPNs exhibited a remarkable sensitivity to stretch, displayed scaled responses to submicrometric membrane indentations, and sustained responses to repetitive mechanical stimuli, consistent with the high mechanical sensitivity of rodent PNs.^[^ ^26^, ^32^, ^42^, ^43^, ^44^ ^]^ Conversely, iLTMRs exhibited lower mechanical thresholds for action potential firing, had reduced sensitivity to small changes in the depths of mechanical probe indentations, and displayed desensitization to repeated mechanical stimulation. Interestingly, this desensitization is similar to that observed in rodent unmyelinated LTMRs (C‐LTMRs),^[^ ^2^, ^45^, ^46^ ^]^ but contrasts with rodent heavily myelinated LTMRs (Aß‐LTMRs) that do not display desensitization upon repeated stimulation.^[^ ^1^ ^]^ A current challenge is to precisely determine the mechanosensory subgroups of iPNs and iLTMRs without the addition of myelination and/or innervation to end organs. Future studies to address this limitation may include conducting single‐cell patch‐sequencing analyses^[^ ^3^, ^47^ ^]^ and co‐culturing induced neurons with end organs (e.g., muscle vs Meissner corpuscle) and glial cells, which may be necessary to further specify induced neurons into subgroups of PNs and LTMRs. The functional differences observed between iPNs and iLTMRs suggest their specialisations may be intrinsically defined prior to their innervation to end organs and potentially independent of the microenvironment. However, it is important to note that in vivo, the interaction of mechanosensory neurons with end organs and Schwann cells is critical for further defining and regulating responses to mechanical stimuli.^[^ ^48^, ^49^, ^50^, ^51^ ^]^ Indeed, subtypes of sensory Schwann cells directly contribute to regulating the sensitivity and thresholds of mechanoreceptors required for low‐threshold touch and nociceptive pain.^[^ ^52^, ^53^ ^]^ Furthermore, LTMR axon protrusions into skin tissue form adherens junctions with the relevant end organs (e.g., Meissner Corpuscle vs Lanceolate ending), which serve as anchor points that, following mechanical stimulation, activate PIEZO2 and allow the neuron axons to detect changes based on the end organ they innervate.^[^ ^48^ ^]^ In addition, within duck Pacinian and Meissner corpuscles, both LTMRs and Lamellar cells respond to mechanical stimuli with distinct responses to mechanical stimulation relying on cell‐cell interactions.^[^ ^50^, ^51^, ^54^ ^]^ The human model established in this work provides an ideal platform to establish co‐culture systems to investigate how interactions between mechanosensory neurons with end organ cell types and/or glia may shape and fine‐tune their responses to mechanical stimulation. Overall, the findings from this study highlight the significance of the molecular composition of iPNs and iLTMRs that govern their unique responses, which then may be further modulated in the presence of end organs needed for their further specification into PN and LTMR subgroups.

Mechanosensation is essential for everyday life, however, the exact molecular mechanisms detecting, regulating, distinguishing, and transducing different sensory stimuli between the mechanosensory neuron subtypes are still unclear. By providing an in‐depth profile of human iPNs and iLTMRs, we determined that the induced neurons had differences in the excitability, responses to mechanical stimulation, and the complement of ion channels expressed. PIEZO2 is the major channel mediating mechanosensation in iPNs and iLTMRs, as its knockdown ablated the response to mechanical stimulation, however, it did not account for the differences between the two populations. It needs to be noted that knock‐down of ion channel/s may result in other in‐direct effects on cellular functionality. Future studies would involve complementary approaches to silence PIEZO2 as well as discover the molecular determinants underpinning the functional differences observed between iPNs and iLTMRs. Previous studies have highlighted the significant role of ELKIN1 in mechanosensation for both rodent and human DRG sensory neurons,^[^ ^55^ ^]^ and similar to other MA channels, expression of *ELKIN1* was comparable between iPNs and iLTMRs. The differential expression of ion channel isoforms, as observed with PIEZO2 in iPNs and iLTMRs, may underpin the functional signatures of each mechanosensory subtype. Additionally, the functional differences may be due to differences in the expression and proportions of voltage‐gated ion channels,^[^ ^4^, ^28^, ^56^ ^]^ mechanosensitive channel auxiliary subunits and modulators,^[^ ^30^, ^31^, ^32^ ^]^ and in the lipid bilayer membrane composition and tension,^[^ ^57^, ^58^ ^]^ which can enhance, modulate, or reduce the response to mechanical stimulation in mechanosensory subtypes. The differences in excitability and response to mechanical stimuli are likely due to the interplay of multiple mechanisms, which together dictate the fine‐tuned differences in mechanical stimulation between human mechanosensory subgroups. Future investigations utilizing iPNs and iLTMRs in co‐culture systems and/or for interrogation of candidate proteins involved in mechanosensory function will offer deeper insights into the intricate mechanisms governing human DRG sensory mechanobiology.

Loss or dysregulation of mechanosensory neuron functioning results in a range of peripheral neuropathies that can cause chronic pain, ataxia, and/or a loss of touch, bladder, stomach, and sexual sensations. By providing a comprehensive functional overview of the generated mechanosensory neurons, these cultures facilitate the screening of potential mechanosensitive modulators, ion channels, and lipid profiles. This is necessary for developing therapeutic strategies for peripheral neuropathies and to provide a greater understanding of the intrinsic cellular mechanisms within sensory neuron mechanosensation and how this can be dysregulated in peripheral neuropathies. This approach can shed light on why specific mechanosensory neurons are impacted in certain diseases, whereas others remain unaffected, such as the involvement of PNs in Friedreich's ataxia^[^ ^59^, ^60^ ^]^ and LTMRs in inflammation,^[^ ^61^, ^62^ ^]^ and Autism Spectrum Disorder.^[^ ^63^, ^64^ ^]^ Additionally, since PIEZO2 has a widespread role in mechanosensation, it is not an ideal drug target. Alternatively, mechanosensitive channel modulators^[^ ^30^, ^65^ ^]^ may provide excellent candidates for the development of compounds that target specific mechanosensory neurons, such as LTMRs, but do not alter the function of the other cell types. Future work utilising activators, inhibitors, and knockdown of modulatory proteins could provide further insight into the mechanisms regulating mechanosensitivity in iPNs and iLTMRs, which in turn could identify potential targets for therapeutic intervention.

## 4 Conclusion

Whilst great progress has been made in generating neuronal populations from hPSC, there remains the challenge of modeling their unique functional properties. This study is one of the first to address this gap by describing an efficient approach for deriving mechanosensory subpopulations from hPSC and, importantly, reveal their distinct functional responses to mechanical stimulation in the absence of end‐organs. The models of human mechanosensory subtypes described in this study, along with their unique functional characteristics, provide a novel platform to advance knowledge of human mechanosensory physiology in healthy and disease states.

## 5 Experimental Section

### Lentiviral Production

The lentiviral expression vectors used in this study include pLV‐TetO‐eGFP‐PuroR (GFP control vector), pLV‐TetO‐hNGN2‐hRUNX3‐GFP‐PuroR (NGN2 + RUNX3 expression vector), and pLV‐TetO‐hNGN2‐hSHOX2‐GFP‐PuroR (NGN2 + SHOX2 expression vector) (Figure S1, Supporting Information), which were designed in‐house using the pLV‐TetO‐hNGN2‐eGFP‐PuroR (Addgene #79823) as a backbone. The designed lentiviral vectors were produced by GenScript. HEK293T cells were maintained at 37 °C 5% CO2 in DMEM/F12 5% Foetal Bovine Serum (FBS) (SFBS – F, Interpath). For lentiviral production, HEK293T cells were passaged with Accutase (#00‐4555‐56, ThermoFisher) and were seeded at a density of 5000 000 cells/T75 flask. Lentiviral particles were produced 24 h after seeding (90–100% confluence) by co‐transfecting 12 µg of the plasmid encoding for the expression vector of interest, or 12 µg of the reverse tetracycline transactivator vector FUW‐M2rtTA (#20342, Addgene), with the lentiviral packaging plasmids: 6 µg pMDL (#12251, Addgene), 3 µg vSVG (#8454, Addgene), and 3 µg RSV (#12253, Addgene), using polyethyleneimine (PEI) (408727, Sigma) at a ratio of 3:1 PEI:DNA in Opti‐MEM (#31985062, Life technologies). The transfection media was replaced with DMEM/F12 5% FBS 6 h post‐transfection. Viral particles were collected at 24, 48, and 72 h post‐transfection and were filtered (0.45 µm pore size) and then centrifuged at 23 500 rpm for 2.5 h, at 4 °C. The pelleted viral particles were resuspended in PBS+/+ at a 200x enrichment (170 µL from 1 x T75 flask), aliquoted, and stored at −80 C until use.

### hPSC Culture

All experiments were approved by the University of Wollongong Human Ethics Committee (2020/450 and 2020/451) and the University of Wollongong Institutional Biosafety Committee (GT18/03, GT19/08, GT19/09, and IBC2108). The hPSC line H9 (WA09, WiCell) was maintained on vitronectin XF (#07180, STEMCELLTM Technologies) coated T25 flasks using TeSR‐E8 media (#5990, STEMCELLTM Technologies), at 37 °C in a humidified incubator at 5% CO2. Media changes were conducted every 1–2 days depending on confluence, and hPSCs were gently passaged, using 0.5 mm EDTA/DPBS−, when cultures reached a confluence of 60–70%

### hPSC Differentiation

hPSC differentiation to sensory neurons was based on previously published methods.^[^ ^15^ ^]^ Generation of sensory neurons involved the stepwise differentiation of hPSCs to caudal neural progenitors (CNPs), to neural crest spheres, to migrating neural crest cells, and finally to sensory neurons, as described below (Figure 1A). Briefly, hPSCs were seeded as single cells at a density of 20 000 cells, in organ culture dishes (60 x 15 mm, #353037, Corning) previously coated with 10 µg mL^−1^ laminin/PBS (#23017015, ThermoFisher) for 24 h, at 4 C, in TeSR‐E8 supplemented with 10 µm Y‐27632. Following 24 h (day 1), the media was replaced with Neural Induction Media (NIM) (components outlined in Table S10, Supporting Information), supplemented with 3 µm CHIR99021 (SML1046, Sigma), and 10 µm SB431524 (#72234, STEMCELL Technologies). A full media change was repeated on day 3 using NIM supplemented with 3 µm CHIR99021 and 10 µm SB431524. Neurospheres were generated by harvesting day 5 CNPs. On day 5, CNPs were gently lifted using 0.5 mm EDTA/DPBS^−^ and scraping and then resuspended in Neuronal Media (NM) (components outlined in Table S10, Supporting Information), supplemented with 20 ng mL^−1^ FGF2 (#78003, STEMCELL Technologies) and 10 ng mL^−1^ BMP2 (RDS355BM010, In Vitro Technologies). The resuspended cell clumps were plated into ultra‐low attachment U‐bottom 96‐well plates (100 µL well^−1^) (CLS7007, Sigma) and the plates were centrifuged at 200 x g for 4 min. Neurosphere formation could be observed after 24 h. On day 8, 50 µL well^−1^ of NM supplemented with 20 ng mL^−1^ FGF2 and 10 ng mL^−1^ BMP2 was added, and half‐media changes were conducted every 3rd day. After 7 days as neurospheres (differentiation day 12), the neurospheres were collected and plated for differentiation to sensory neurons. To enrich for migrating neural crest cells, on differentiation day 12 the neurospheres were plated as whole spheres on 12‐ or 13‐mm glass coverslips, previously coated with 10 µg mL^−1^ Poly‐D‐Lysine (P6407, Sigma–Aldrich) and 10 µg mL^−1^ laminin, with NM supplemented with 10 µm Y‐27632. On day 14 (48 h after neurosphere plating), the neurospheres were removed using a P200 pipette, leaving behind the migrating neural crest cells. The neural crest cells were then transduced with 1–2 µL mL^−1^ of lentiviral particles containing either pLV‐TetO‐eGFP‐PuroR, pLV‐TetO‐hNGN2‐hRUNX3‐GFP‐PuroR or pLV‐TetO‐hNGN2‐hSHOX2‐GFP‐PuroR sequence and 1–2 µL mL^−1^ FUW‐M2rtTA lentiviral particles for 16 h in NM supplemented with 10 µm Y‐27632, 10 ng mL^−1^ BDNF (78005, STEMCELL Technologies), 10 ng mL^−1^ GDNF (78058, STEMCELL Technologies), 10 ng mL^−1^ NT‐3 (78074, STEMCELL Technologies), and 10 ng mL^−1^ ß‐NGF (78092, STEMCELL Technologies). To remove any virus and to induce transcription factor expression, a full media change was conducted on the following day (differentiation day 15), containing 1 µg mL^−1^ doxycycline (D9891, Sigma) NM supplemented with 10 µm Y‐27632, 10 ng mL^−1^ BDNF, 10 ng mL^−1^ GDNF, 10 ng mL^−1^ NT‐3 and 10 ng mL^−1^ ß‐NGF. Transcription factor expression was induced by the addition of doxycycline for 96 h (differentiation days 15–19). To select successfully transduced cells, 1 µg mL^−1^ puromycin (73342, STEMCELL Technologies) was added for 48 h (day 17–19). To mature the progenitors into sensory neurons, media changes of NM supplemented with 10 µm Y‐27632, 10 ng mL^−1^ BDNF, 10 ng mL^−1^ GDNF, 10 ng mL^−1^ NT‐3 and 10 ng mL^−1^ ß‐NGF were conducted every 2–3 days. To functionally mature the sensory neurons and mimic the nervous system's extracellular environment BrainPhys Neuronal Medium (BNM) (Components outlined in Table S10, Supporting Information) was phased into the NM beginning on differentiation day 22 (25:75, 50:50, 75:25, 100:0 BNM: NM), with the same concentrations of growth factors as specified above in each media change. Between differentiation days 25–27, proliferating cells in the culture were removed using 2.5 µm cytosine β‐D‐arabinofuranoside (AraC) (C1768, Sigma) for 48 h. If neurons began to detach or cluster together, 1 µg mL^−1^ laminin was supplemented into the media to promote reattachment. The neurons were matured until day 34 and were then fixed for immunocytochemistry or harvested for total RNA extraction. Calcium imaging and patch‐clamp functional analyses were performed between days 34–48.

### Immunocytochemistry

When cultures reached the required stage for staining, the cells were washed with PBS 3 times and fixed with 4% paraformaldehyde/PBS for 20 min, at room temperature, and then PBS washed 3 times. Cells were permeabilised with 0.1% triton/PBS for 10 min and then blocked in blocking buffer (10% donkey serum/PBS (D9663, Sigma)) for 1 h at room temperature. The cultures were incubated with the appropriate primary antibody (Table S11, Supporting Information) in blocking buffer overnight at 4 °C. Following the overnight incubation, the coverslips were washed with PBS 3 times for 5 min and then incubated with the appropriate secondary antibody (Table S11, Supporting Information) in the dark in blocking buffer for 1 h at room temperature. The secondary antibody solution was removed, and the samples were washed 3 times for 5 min in PBS and counter‐stained with 1:1000 DAPI (D9542, Sigma) for 15 min. DAPI stain excess was removed after 3 repetitive 5 min PBS washes. The coverslips were mounted with a drop of ProLong Gold Antifade Mountant (P36934, Life Technologies Australia) onto microscope slides (MENSF41296P, ThermoFisher). Stained microscope slides were stored at 4 °C in a dark microscope slide container until imaged. Images were taken using a Leica confocal SP8 microscope and exported using ImageJ (FIJI) software. Cell counts were performed using the cell count FIJI tool. Colors used in the final figure images were colorblind safe combinations.

### Protein Harvesting and Quantification

Protein was extracted in RIPA buffer (R0278, Sigma) supplemented with protease inhibitor cocktail (P8340, Sigma). The cell lysate was centrifuged at 12 000 rpm for 20 min at 4 °C, and the supernatant was collected and stored at −80 °C. The total protein concentration was determined via a Detergent‐compatible (DC) colorimetric assay (5000112, Bio‐Rad) following the manufacturer's instructions. For Western blot analysis, the protein samples (5 µg) were resuspended in sodium dodecyl sulfate‐polyacrylamide gel electrophoresis (SDS‐PAGE) loading buffer (5% v/v b‐mercaptoethanol (M7154, Sigma), 2x Laemmli ([0.01% v/v bromophenol blue, 25% v/v glycerol, 2% v/v SDS, 62.5 mm Tris‐HCl, pH 6.8]), denatured at 95 °C for 5 min and then placed on ice. Samples were separated by SDS‐PAGE electrophoresis at 100 volts (V) for 1 h on 4–20% Criterion TGX Stain‐Free Protein Gels (1656001, Bio‐Rad) and 1x SDS‐page running buffer (192 mm glycine, 3.5 mm SDS, 25 mm Tris‐hydroxymethyl‐methylamine). Following protein separation, the 4–20% Criterion TGX Stain‐Free Protein Gel (M3148, Bio‐Rad) was activated by a GelDoc XR+ (BioRad). Protein samples were transferred onto a 0.45 µm pore polyvinylidene difluoride (PVDF) membrane (IPVH00010, Millipore), previously activated in cold 100% methanol, using a Criterion blotter (1704070, Bio‐Rad) at 100 V in transfer buffer (192 mm glycine, 20% v/v methanol, 25 mm tris‐hydroxymethyl‐methylamine) for 1.5 h. Following transfer, the membranes were washed in 0.05% Tween (P1379, Sigma) in PBS (PBST) on a rocker and imaged using GelDoc XR+ for total protein. Membranes were blocked with 10% milk/PBS rocking for 1 h, at room temperature. Membranes were incubated with the appropriate primary antibody (Table S12, Supporting Information) in 10% milk by rocking for 16 h at 4 °C. The primary antibodies were removed by washing 4 times with PBST over 20 min. The membranes were incubated with the appropriate secondary antibody (Table S12, Supporting Information) rocking for 1 h at room temperature. The membranes were then washed 4 times with PBST and then incubated in the dark for 5 min with Clarity Western ECL substrate (1705060, Bio‐Rad), and imaged using the Amersham Imager 600 (GE Industries, UK).

### RT‐qPCR and Bulk RNA Sequencing

RNA was purified using the PureLink RNA Mini Kit (12183025, ThermoFisher) according to the manufacturer's instructions. RNA concentration and quality were assessed using a Nanodrop spectrophotometer and a Bioanalyzer (Agilent). Using up to 1 µg of RNA per reaction, genomic DNA was removed, and the RNA was reverse transcribed into cDNA using the iScript gDNA Clear cDNA Synthesis Kit (1725035, Bio‐Rad), as per the manufacturer's guidelines. RT‐qPCR was conducted using the PowerUP SYBR green master mix (A25778, ThermoFisher) in a QuantStudio 5 real‐time PCR system (Applied Biosystems) using the fast run mode settings, following the manufacturer's instructions. RNA samples that passed the quality control check (A260:280 >= 2.0, RNA and integrity number > 7.0) were utilised for sequencing. Library preparation and RNASeq analyses were performed as a service from the Garvin Institute for Medical Research (Genome One) (2x 100 base pairs, 30 million read pairs)

### Bulk RNA Sequencing Analysis

The initial RNAseq processing involved the utilization of DRAGEN RNA Pipeline 3.7.5. Following adapter trimming, RNAseq reads with a Phred Quality score greater than 20 were retained and preprocessed. The input reads ranged from 124 to 190 million, with paired proportions falling within the range of 94.7–96.3% and median insert sizes ranging from 115 to 137. During the parameter selection phase, the GRCh38 reference was chosen, excluding alternative contigs and including decoy, while the gencode_grch38.v32.annotation.gtf was employed for RNA annotation purposes. The DRAGEN RNA pipeline utilized the DRAGEN RNA‐Seq spliced aligner. After obtaining the raw count matrix, rows with zero expression were removed. ComBat_seq^[^ ^66^ ^]^ normalization was applied to correct potential biases due to batches of sub‐cultures. Further, Gene differential expression analysis was performed using Deseq2.^[^ ^67^ ^]^ The average data from 3 biological replicates was presented as the log2 Transcripts per Million (Log2TPM).

### Calcium Imaging

Sensory neuron cultures were used for calcium imaging between differentiation days 34–48, once they reached the desired maturity. Neurons were incubated for 40 min at 37 °C and 5% CO_2_ in Calcium imaging Bath Solution (CBS) containing (in mm); 160 NaCl, 2.5 KCL, 5 CaCl_2_, 1 MgCl_2_, 10 HEPES, and 5 Glucose (pH 7.4, 320 mOsm per kg), supplemented with 6 µm Fura‐2AM (F1221, ThermoFisher), and 0.04% Pluronic F‐127 (P2443, Sigma). Following incubation, cultures were washed with CBS and transferred to a Warner Series 20 Chamber (Warner Instruments, USA) mounted on an imaging platform. Neurons were perfused continuously with CBS at 1 mL min^−1^ using a MasterFlex C/L peristaltic pump (MasterFlex, Germany) throughout imaging. Calcium imaging was conducted in the dark using a Leica DMi8 epi‐fluorescence microscope (Leica Microsystems Pty Ltd., Lane Cove West NSW, Australia) with a dichromatic filter enabling dual‐wavelength excitation at 340 and 380 nm. Images were acquired every 0.7 s using a 20x dry objective, 2x2 binning, 100 ms exposure time, and 16‐bit resolution. The Leica LAS‐X calcium imaging software was used for acquisition and analysis.

Each experiment began with 2–4 min of CBS perfusion to establish a fluorescence baseline. Neurons were then exposed to CBS containing the required agonist, followed by depolarization with 60 mm KCl. Bath solution washes were performed between agonist and KCl treatments. Agonists included 100 µm Allyl isothiocyanate (AITC; 377430), 1 µm Capsaicin (M2028), 250 µm Menthol (M2772), and 1 µm GSK1702934A (SML2323) (all from Sigma).

### Calcium Imaging – Stretch

For the isotropic stretch experiments, hPSCs were differentiated and transduced as described previously in *hPSC differentiation*. Neural crest cells were plated onto PDMS *IsoStretcher* chambers^[^ ^33^ ^]^ and allowed to mature. Stretch calcium imaging was performed under static bath conditions using CBS, with modifications to the imaging protocol. The *IsoStretcher* ^[^ ^34^ ^]^ device was calibrated via a LabVIEW‐to‐Arduino interface, and chambers were mounted into the *IsoStretcher* actuator. Imaging was conducted using a Leica DMi8 microscope and LAS‐X software with dual excitation at 340 and 380. Images were captured every 0.45 s, with 20x dry magnification, and imaging parameters set at 2x2 binning, 50 ms exposure, mercury lamp setting 2, and 16‐bit resolution. To account for the z‐plane shifts, the neurons were first imaged for 30s in the initial focal plane, followed by 30s in the shifted z‐plane. Neurons were then isotropically stretched by 10% (radial increase of the chamber) using the LabVIEW‐to‐Arduino interface software. Three minutes post‐stretch, a final maximal depolarizing stimulus was applied by adding KCl to a final concentration of 60 mm. For the gadolinium experiments, chambers were pre‐incubated with 300 µm gadolinium (Gadolinium (III) chloride hexahydrate; 13450‐84‐5, Sigma) for 5 min before imaging and stretch. In calcium‐free controls, CBS was replaced with a calcium‐free CBS (10 mm EGTA, 162 mm NaCl, 2.5 mm KCl, 1 mm MgCl_2_, 10 mm HEPES, 5 mm Glucose; pH 7.4, 320 mOsm per kg), and the imaging protocol was carried out as described above.

### Calcium Imaging Analysis

Calcium imaging data were analysed using LAS‐X calcium imaging software. Traces were generated by calculating the change in the 340/380 fluorescence ratio, subtracted by the baseline fluorescence of each cell. For the stretch experiments, due to some soma and neurites shifting in and out of the field of view once the stretch occurred, the baseline fluorescence of each cell was taken as the average fluorescence intensity of 20 s before KCl addition. The maximum of the responses to the agonist and KCl were calculated using a custom script written on Python software available at: https://doi.org/10.5281/zenodo.7460899. The percentage response was calculated by:

(1) $$\begin{array}{ccc} & & \mathrm{Percentage}\;\mathrm{response}\\ & & \;=\left(\mathrm{maximum}\;\mathrm{of}\;\mathrm{agonist}\right)÷\left(\mathrm{maximum}\;\mathrm{of}\;\mathrm{KCl}\right)*100 \end{array}$$

To determine the proportion of responsive cells, a signal‐to‐noise cut‐off threshold of 2% (of maximal response) was set as values below the threshold were indistinguishable from noise and thus classified as “no response”.

### Electrophysiology

Whole‐cell patch‐clamp recordings were performed at room temperature (20–24 °C) using an inverted Nikon microscope and a MultiClamp 700B Amplifier. Signals were digitized using a Digidata 1440 interface and acquired with pClamp11 software (Molecular Devices, San Jose, CA, USA). For current clamp experiments, the bath solution contained (in mm): 135 mm NaCl, 2 mm CaCl_2_, 2 mm MgCl_2_, 5 mm KCl, 10 mm glucose, 10 mm HEPES, adjusted to pH 7.4, with an osmolality of 315± 5 mOsm per kg. For voltage‐clamp experiments, the bath solutions were tailored according to the ion channel being examined. For K^+^ currents (I_K_), the bath solution above was supplemented with 1 µm tetrodotoxin citrate (TTX; Abcam, Melbourne, Australia) to block Na^+^ currents. Na^+^ currents (I_Na_) were isolated using a bath solution containing (in mm): 110 NaCl, 2 CaCl_2_, 2 MgCl_2_, 30 TEA‐Cl, 10 D‐Glucose, and 10 HEPES, pH 7.3. To isolate Ca^2+^ currents (I_Ca_), the extracellular solution contained (in mm): 140 TEA‐Cl, 10 CaCl_2_, 1 MgCl_2_, 10 HEPES, 10 D‐Glucose, pH 7.3. Patch pipettes (borosilicate glass; World Precision Instruments, USA) were pulled using a P‐97 Flaming/Brown micropipette puller (Sutter Instruments), fire polished and filled with intracellular solution containing (in mm):140 K‐gluconate, 10 NaCl, 2 MgCl_2_, 10 HEPES, 5 EGTA, pH 7.2, osmolality 295± 5 mOsm per kg, and had resistances between 2–4 mΩ. Whole‐cell recording configuration was acquired under voltage‐clamp mode. Series resistance was compensated by ≥ 60%, and whole‐cell currents were sampled at 100 kHz and filtered at 10 kHz. Neuronal excitability was assessed under current‐clamp mode. Action potentials were evoked using 1 s current steps ranging from −150 to 150 pA in 10 pA increments. Ion channel modulators (Gadolinium, ICA 121431, Hm1a, ZD7288) were applied via whole‐bath perfusion.

### Mechanical Stimulation

Mechanical stimulation of neurons was delivered using a fire‐sealed borosilicate glass pipette (“probe”) filled with intracellular solution. The probe was mounted at a 45° angle relative to the glass coverslip and positioned using a PatchStar micromanipulator (Scientifica), controlled by LinLab 2 software. The standard mechanically‐activated (MA) stimulation protocol consisted of ten sequential 0.1 µm indentations, delivered at 2‐s intervals (0.5 Hz) with increasing indentation depths from 0.1 µm to 1.0 µm (∆ = 0.1 µm). Recordings were conducted in gap‐free voltage‐clamp or current‐clamp mode. Any variations in the stimulation protocol were described in the respective figure legends.

### siRNA Transfection or Knockdown of PIEZO1 and PIEZO2

Neurons were transfected using Accell SMARTpool siRNA (knockdown day 1) with either 1 µm non‐targeting siRNA (D‐001960‐01‐20, Accell, Horizon Discovery Group Company), 1 µm human SMARTpool PIEZO1 siRNA (E‐020870‐00‐050, Accell, Horizon Discovery Group Company) or 1 µm PIEZO2 (E‐013925‐00‐0050, Accell, Horizon Discovery Group Company) in BrainPhys Media supplemented with 10 µm Y‐27632, 10 ng mL^−1^ BDNF, 10 ng mL^−1^ GDNF, 10 ng mL^−1^ NT‐3 and 10 ng mL^−1^ ß‐NGF. Following 48 h transfection (knockdown day 3), a full media change containing fresh siRNAs was conducted. The neurons were whole‐cell patch clamped 72 h post initial siRNA transfection (KD day 4). To confirm siRNA knockdown, neurons were harvested for RNA, and RT‐qPCR was conducted. To determine the mechanical response following transfection, the neurons were whole‐cell voltage clamped using borosilicate glass patch pipettes fire polished to a resistance between 2–4 mΩ and filled with CsF‐intracellular buffer (110 mm CsF, 30 mm CsCl, 10 mm NaCl, 2 mm MgCl_2_, 10 mm HEPES, and 5 mm EGTA, pH 7.2, osmolarity 295± 5 mOsm per kg).

### Electrophysiology Analysis

Electrophysiological traces and action potentials were analyzed using Clampfit version 11.1.0.23. Rheobase was defined as the minimum current required to evoke a single action potential. Action potential parameters were quantified from the rheobase‐evoked action potential. MA currents were pre‐processed using in‐built Clampfit functions by (1) baselining to the average current level recorded 30 ms before stimulation and (2) applying a low‐pass Gaussian filter (cut‐off 770 Hz; 13 coefficients). Mechanically activated current recordings were exported as CVS files, and peak amplitudes were extracted using a custom Python script available at https://doi.org/10.5281/zenodo.7460899. To calculate the mean current decay time constant (τ), MA currents were recorded under gap‐free voltage‐clamp conditions using a 0.5 µm membrane probe indentation (100 ms duration). MA currents were fit to a single or multi‐exponential decay function:

(2) $$f\left(t\right)=\sum_{i=1}^{n}A_{i}e^{-t/\tau i}+C$$

Mechanical activation kinetics were assessed by normalizing the induced mechanical current amplitude (I_MA_) to the maximal current response (I_MAX_) for each neuron. The resulting I_MA_/I_MAmax_ values were fit to a Boltzmann sigmoid function with constraints (top = 1, bottom = 0), yielding a “mechanical dependence of activation” curve. From this, the indentation displacement required to reach 50% activation (Indent_50_) and the slope of the curve (mechanosensitivity) were determined.

Voltage‐dependent activation and steady‐state inactivation (SSI) of ionic currents were analyzed by fitting a modified Boltzmann equation:

(3) $$\mathrm{Activation}:\;G=1-1/\left(1+\exp\;\left(Vm-V_{0.5/}ka\right)\right)$$

(4) $$\mathrm{SSI}:I=1/\left(1+\exp\;\left(Vm-V_{0.5/}ka\right)\right)$$

where I is current, G is conductance, Vm is pre‐pulse membrane potential, V_0.5_ is the half‐maximal activation/inactivation potential, and ka is the slope factor.

### Statistical Analysis

All statistical analysis was performed and presented using GraphPad Prism 9 unless stated otherwise. Data were presented as the mean ± standard error of the mean (SEM). Groups were compared using either Student's *T*‐test or One‐Way Analysis of Variance (ANOVA) with Tukey post‐hoc test to determine statistical significance. The specific statistical method and number of replicates were specified in the relevant figure legends and tables.

## Conflict of Interest

The authors declare no conflict of interest.

## Supporting information



Supporting Information

## Data Availability

The data that support the findings of this study are available from the corresponding author upon reasonable request.
