# Supplementary material for: Induced Proprioceptor and Low‐Threshold Mechanoreceptor Neurons Derived from Human Pluripotent Stem Cells Exhibit Distinct Functional Mechanosensory Properties
Source: Adv Sci (Weinh). 2025 Dec 9;13(6):e12413. doi: 10.1002/advs.202512413 (PMC12866804; doi:10.1002/advs.202512413)
Supplement: Supplementary file 1 — Supporting Information [file ADVS-13-e12413-s001.docx]

**Supporting Information**

Supporting Information is available from the Wiley Online Library or from the author.

Supplementary Text

Figs. S1 to S8

Tables S1 to S12

**Supplementary figures**

**
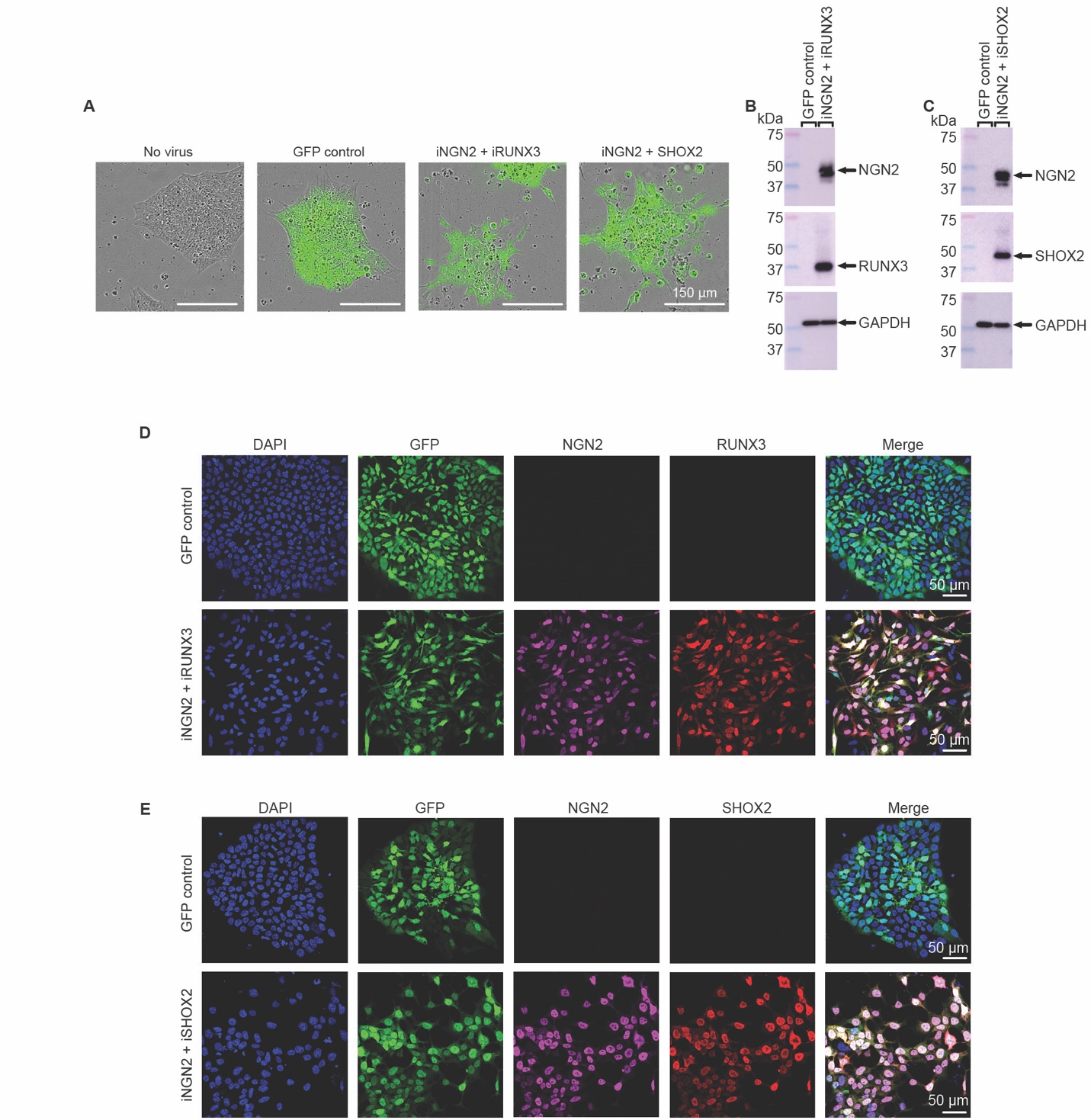
**

**Fig. S1: Validation of GFP, NGN2 and either RUNX3 or SHOX2 expression in hPSCs.** hPSCs were transduced with lentiviral viral particles containing the reverse tetracycline transactivator (rTTA) and either pLV- TetO-eGFP-PuroR (GFP control), pLV-TetO-hNGN2-hRUNX3-GFP-PuroR (iNGN2 + iRUNX3) or pLV- TetO-hNGN2-hSHOX2-GFP-PuroR (iNGN2 + iSHOX2). Viral particles were removed and doxycycline was administered 24 h following transduction. hPSCs were harvested for Western blotting or immunocytochemistry after 96 h of doxycycline administration. (A) Representative images of transduced cultures that confirm the expression of GFP after 96 h of doxycycline incubation. Western blots of hPSC protein lysates probed for (B) NGN2 and RUNX3 or (C) NGN2 and SHOX2 protein expression. (D) Representative immunocytochemistry images showing the cellular co-localisation of GFP (*green*) with NGN2 (*magenta*) and RUNX3 (*red*) or (E) with NGN2 (*magenta*) and SHOX2 (*red*). Nuclei are shown in blue. Scale bars = 50 μm.


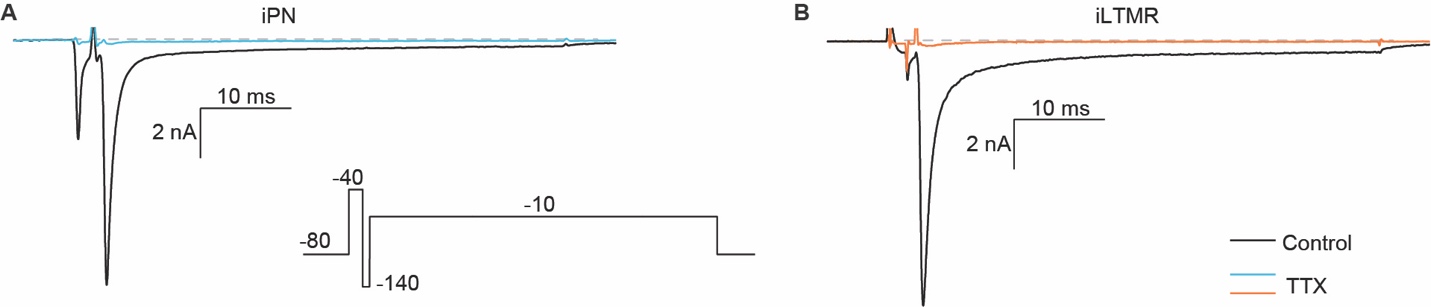


**Fig. S2: TTX inhibition of I_Na_ in iPN and iLTMR.** Representative Na^+^ currents recorded from (**A**) iPN and (**B**) iLTMRs in the absence (control) and presence of 300 nM TTX. Inset: stimulus protocol (50 ms, -10 mV, Vh -80 mV, 0.1Hz). Scale bars: 2 nA, 10 ms. Numerical data are presented in Table S3.


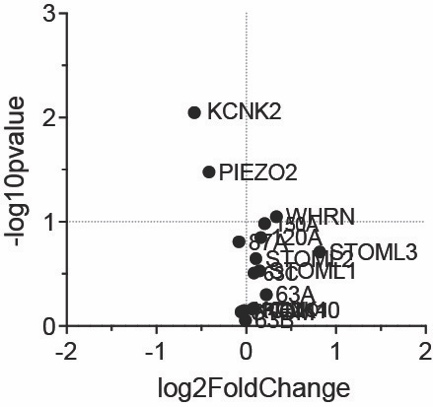


**Fig. S3: Differential gene expression plot comparing mechanosensitivity-associated transcripts between iPNs and iLTMRs.** The major gene transcripts associated with the detection of mechanical sensations by comparing the iPN expression (*right*) against the iLTMR expression (*left*), from bulk RNA sequencing. n = 3 biological replicates.


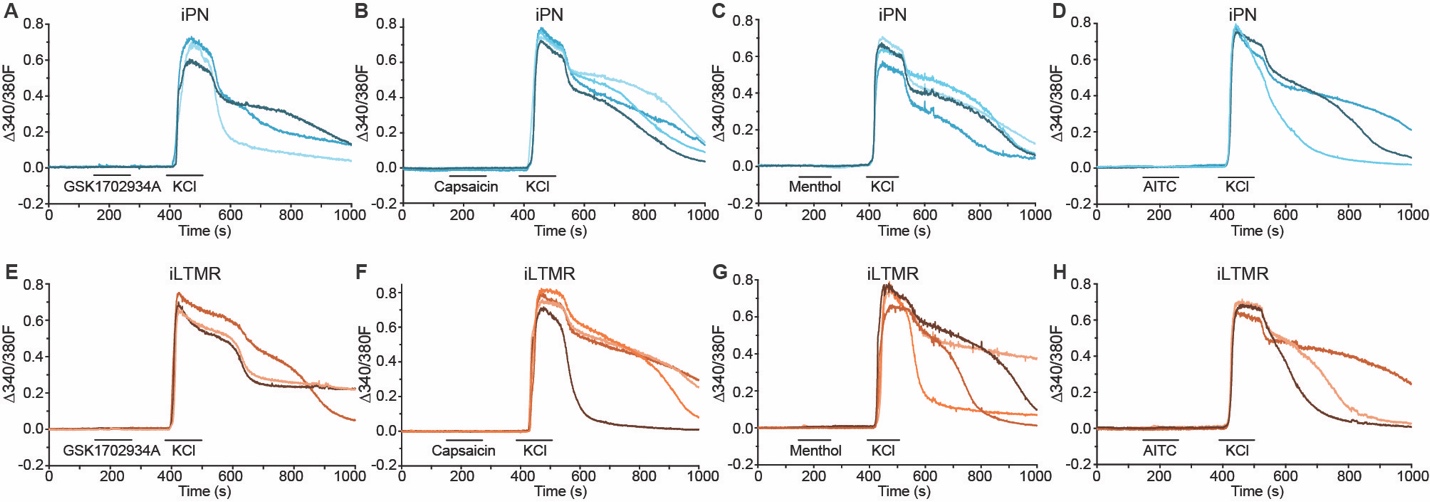


**Fig. S4. Absence of Nociceptive Responses in iPNs and iLTMRs.** Representative live-cell Fura-2 calcium imaging traces of iPNs (*top*) and iLTMRs (*bottom*) in the presence of the following nociceptive agonists: (A, E) 1 µM GSK1702934A (TRPC3/6 activator), (B, F) 1 µM capsaicin (TRPV1 activator), (C, G) 250 µM menthol (TRPM8 activator), and (D, H) 100 µM AITC (TRPA1 activator). Traces show changes in the 340/380 fluorescence ratio (Δ340/380F) measured every 0.7 s, from baseline- in 3 – 4 individual neurons perfused (1 mL/min) with the indicated agonist. Each treatment was followed by 60 mM KCl application, with CBS washes before and after treatments. n > 100 neurons per agonist across 3 biological replicates. Numerical data are provided in Table S4.


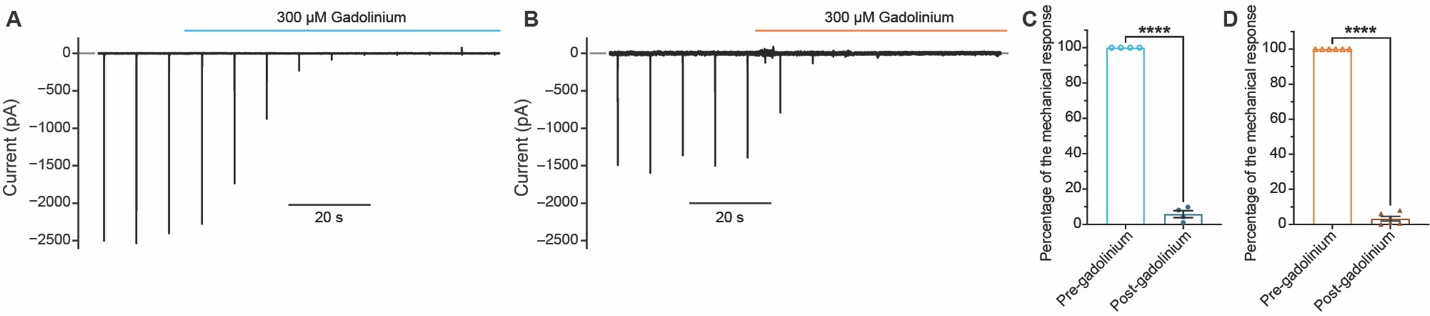


**Fig. S5. Gadolinium inhibits MA currents in iPNs and iLTMRs.** Representative whole-cell voltage-clamp recordings from an (**A**) iPN and (**B**) iLTMR neuron in response to repetitive 0.5 μm membrane probe indentations (100 ms duration), followed by bath application of 300 μM gadolinium in extracellular buffer. (**C**, **D**) Normalised mechanical response (%) before and after gadolinium treatment in (**C**) iPNs and (**D**) iLTMRs, respectively, calculated relative to the average pre-treatment response. Unpaired t-test, ****p < 0.0001. n = 3 – 4 biological replicates; n = 4 – 6 neurons in total. Data are presented as mean ± SEM.


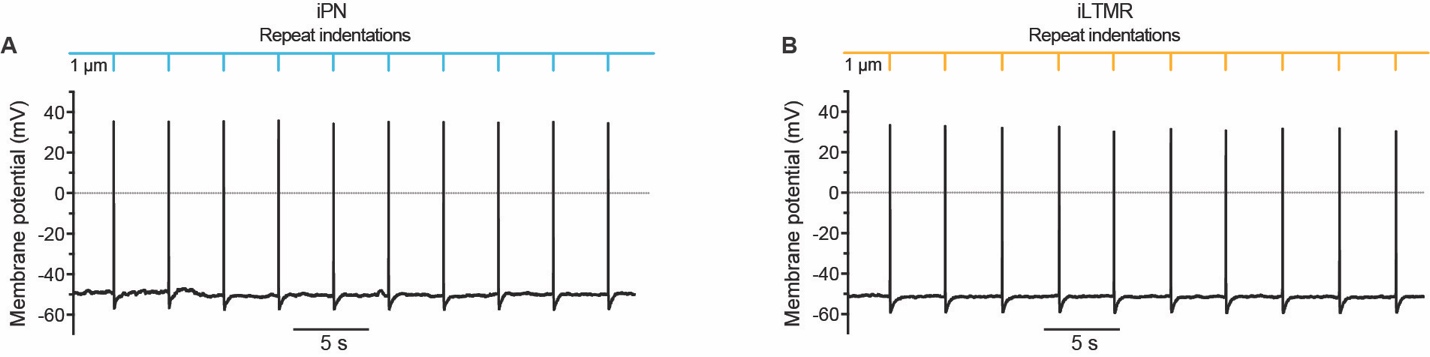


**Fig. S6. iPNs and iLTMRs fire stereotypical action potentials in response to mechanical stimulation.** Representative whole-cell current-clamp recordings from an (A) iPN and (B) iLTMR neuron during repeated 1 µm membrane probe indentations (100 ms duration), showing consistent action potential firing in response to mechanical stimuli.


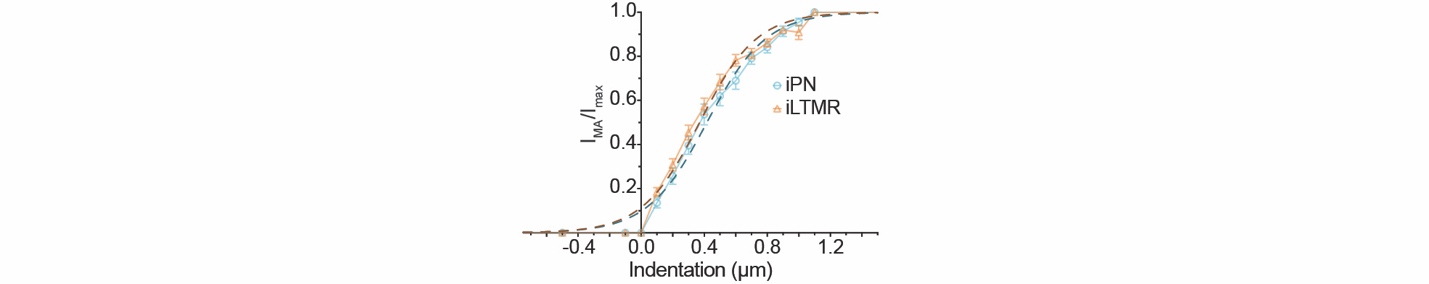


**Fig. S7. I_MA_ activation as a function of membrane indentation.** Normalised mechanically activated I_MA_ derived from data in Fig. 4, plotted relative to the maximum current (I_MAX_) across increasing membrane probe indentation depths. n = 25 – 33 neurons from n = 5 – 8 biological replicates.


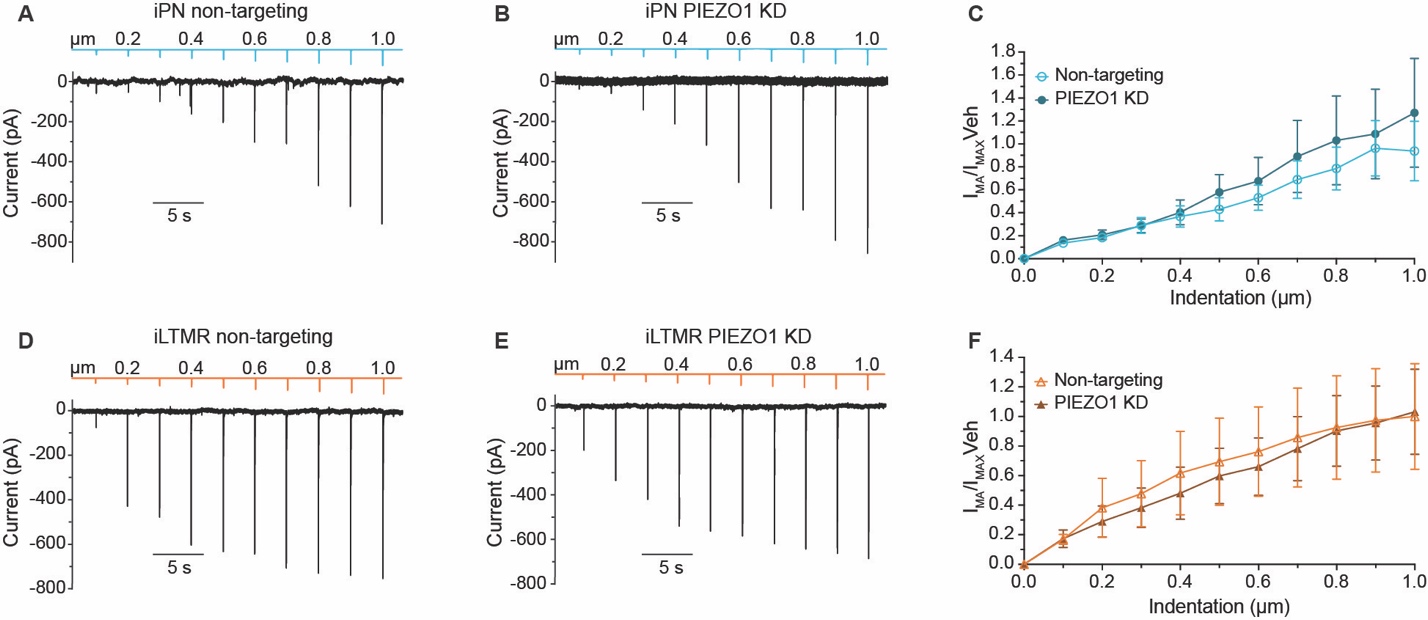


**Fig. S8. PIEZO1 does not contribute to the mechanically activated (MA) current in iPNs and iLTMRs.** Whole-cell voltage-clamp recordings from iPNs and iLTMRs stimulated with increasing increments (0.1 μm) of membrane probe indentation (0 – 1 μm, ∆ 0.1 μm). Representative traces from iPNs transfected with (A) non-targeting siRNA or (B) PIEZO1 siRNA for 96 h. (C) Current density in iPNs normalised to the average response at 1 μm indentation in the non-targeting siRNA group. . Representative traces from iLTMRs transfected with (D) non-targeting siRNA or (E) PIEZO1 siRNA for 96 h. (F) Current density of iLTMRs normalised to the average response at 1 μm indentation in the non-targeting siRNA group. Data are presented as mean ± SEM, n = 15 – 16 neurons from 3 independent siRNA transfections.

**Supplementary tables**

**Table S1. Quantification of BRN3A+ and ISLET1+ cells**

|  | **iPN** | | | **iLTMR** | | |  |
| --- | --- | --- | --- | --- | --- | --- | --- |
|  | **Mean** | **SEM** | **n** | **Mean** | **SEM** | **n** | **Unpaired t-test** |
| BRN3A+ cells (%) | 88 | 2.175 | 3 | 89.37 | 2.628 | 3 | 0.7088 |
| ISLET1+ cells (%) | 84.48 | 6.853 | 3 | 88.59 | 1.11 | 3 | 0.5856 |

^n = biological replicates

Mean percentages ± SEM of BRN3A⁺ and ISLET1⁺ cells in iPN and iLTMR populations, based on immunocytochemistry. Each group includes data from *n* = 3 biological replicates. Statistical comparison was performed using an unpaired *t*-test

**Table S2. Quantification of excitability profiles in iPNs and iLTMRs**

|  | **iPN** | | | **iLTMR** | | |  |
| --- | --- | --- | --- | --- | --- | --- | --- |
|  | **Mean** | **SEM** | **n** | **Mean** | **SEM** | **n** | **Unpaired t-test** |
| Resting membrane potential (mV) | -55.4 | 0.757 | 40 | -55.4 | 0.746 | 42 | 0.9995 |
| Capacitance (pF) | 31.64 | 2.91 | 32 | 28.75 | 2.373 | 45 | 0.4411 |
| Rheobase (pA) | 49.71 | 5.609 | 34 | 52.38 | 5.222 | 42 | 0.729 |
| Number of action potentials at 2 times rheobase | 6.943 | 0.8106 | 35 | 5.865 | 0.7209 | 37 | 0.3226 |
| Maximum action potentials fired | 12 | 1.39 | 36 | 10.5 | 1.24 | 36 | 0.4223 |
| Hyperpolarisation sag ratio at -140pA | 0.3484 | 0.01779 | 37 | 0.2782 | 0.0223 | 38 | **0.0166** |
| Time to peak (ms) | 50.15 | 4.325 | 34 | 68.05 | 5.32 | 42 | **0.0136** |
| Rise time (ms) | 25.66 | 2.654 | 34 | 40.21 | 3.824 | 42 | **0.0039** |
| Action potential half-width (ms) | 2.94 | 0.1985 | 34 | 4.32 | 0.3896 | 42 | **0.0043** |
| Rise slope (mV/ms) | 0.9534 | 0.0964 | 34 | 0.5887 | 0.0715 | 42 | **0.0027** |
| Peak amplitude (mV) | 94.79 | 1.875 | 34 | 95.11 | 1.747 | 42 | 0.9016 |

^n = neurons

Summary of electrophysiological properties measured in iPN and iLTMR neurons. Values are presented as mean ± SEM. Sample sizes (*n*) represent the number of neurons recorded. Statistical comparisons between groups were performed using unpaired *t*-tests.

**Table S3. Quantification of the ionic basis of excitability in iPNs and iLTMRs**

|  | **iPN** | | | **iLTMR** | | |  |
| --- | --- | --- | --- | --- | --- | --- | --- |
|  | **Mean** | **SEM** | **n** | **Mean** | **SEM** | **n** | **Unpaired t-test** |
| I_Nav_ (pA/pF) | -345.5 | 74.6 | 18 | -420.5 | 41.8 | 24 | 0.3572 |
| Na_v_ act V_0.5_ (mV) | -35.6 | 1.7 | 18 | -37.1 | 1.1 | 25 | 0.4352 |
| Na_v_ inact V_0.5_ (mV) | -61.9 | 1.9 | 17 | -60.0 | 1.1 | 17 | 0.4116 |
| Na_v_ TTX block (%) | 99.4 | 0.25 | 6 | 99.4 | 0.1 | 6 | 1 |
| Na_v_ ICA block (%) | 33.8 | 6.5 | 8 | 38.0 | 7.5 | 9 | 0.6789 |
| Na_v_ Hm1a fold change | 2.9 | 0.8 | 8 | 3.1 | 0.7 | 11 | 0.8224 |
| HVA I_Cav_ (pA/pF) | -37.5 | 11.1 | 9 | -23.0 | 4.4 | 13 | 0.5026 |
| LVA I_Cav_ (pA/pF) | -5.0 | 1.1 | 9 | -3.2 | 1.3 | 13 | **0.0058** |
| I_Kv_ (pA/pF) | 128.2 | 11.6 | 35 | 125.2 | 9.8 | 29 | 0.8467 |
| K_v_ act V_0.5_ (mV) | 3.8 | 2.3 | 22 | 5.0 | 1.9 | 32 | 0.7131 |
| K_v_ inact V_0.5_ (mV) | -37.3 | 1.9 | 34 | -35.9 | 1.9 | 29 | 0.6113 |
| I_h_ (pA/pF) | -19.0 | 3.5 | 11 | -17.1 | 2.7 | 10 | 0.6703 |
| I_h_ act V_0.5_ (mV) | -98.4 | 2 | 11 | -101.6 | 1.8 | 9 | 0.2758 |
| I_h_ tau act (ms) | 145.3 | 13.6 | 8 | 200.5 | 19.3 | 8 | **0.0351** |

^n = neurons

Electrophysiological properties related to sodium (Nav), calcium (Cav), potassium (Kv), and hyperpolarization-activated (Ih) currents recorded from iPN and iLTMR neurons. Values are expressed as mean ± SEM, with sample sizes (*n*) indicating the number of neurons analyzed. Statistical comparisons between groups were performed using unpaired *t*-tests.

**Table S4: Quantification of the iPN and iLTMR response to agonists**

|  | **iPN** | | | **iLTMR** | | |  |
| --- | --- | --- | --- | --- | --- | --- | --- |
|  | **Mean** | **SEM** | **n** | **Mean** | **SEM** | **n** | **Unpaired t-test** |
| GSK1702934A (% normalised to KCl) | 1.451 | 0.2422 | 117 | 1.088 | 0.1391 | 144 | 0.1756 |
| Capsaicin (% normalised to KCl) | 0.7645 | 0.06326 | 144 | 0.7404 | 0.1027 | 164 | 0.8469 |
| Menthol (% normalised to KCl) | 0.6908 | 0.04032 | 146 | 0.9303 | 0.1733 | 166 | 0.2052 |
| AITC (% normalised to KCl) | 1.359 | 0.3686 | 119 | 1.091 | 0.1386 | 160 | 0.4511 |

^n = neurons

Mean responses of iPN and iLTMR neurons to various agonists, normalized to the response elicited by 60 mM KCl. Values are presented as mean ± SEM, with *n* indicating the number of neurons analyzed. Statistical comparisons between groups were performed using unpaired *t*-tests.

**Table S5. Quantification of the iPN and iLTMR responses to mechanical stretch**

|  | **iPN** | | | **iLTMR** | | |  |
| --- | --- | --- | --- | --- | --- | --- | --- |
|  | **Mean** | **SEM** | **n** | **Mean** | **SEM** | **n** | **Oneway ANOVA, Tukey multiple comparisons test** |
| Soma stretch  (% normalised to KCl) | 39.45 | 1.021 | 250 | 43.38 | 0.6636 | 410 | iPN soma vs iLTMR soma **0.0061**  iPN soma vs iPN neurite bundle: 0.6951  iLTMR soma vs iLTMR neurite bundle: **0.0314** |
| Neurite bundle stretch  (% normalised to KCl) | 42.15 | 1.696 | 43 | 49.78 | 3.354 | 46 | iPN neurite bundle vs iLTMR neurite bundle: 0.0777 |
|  |  |  |  |  |  |  | **Unpaired t-test** |
| Soma stretch under calcium-free conditions  (∆ 340/380F) | 0.00237 | 0.000347 | 81 | 0.00938 | 0.00407 | 123 | 0.1646 |
| Neurite bundle stretch under calcium-free conditions  (∆ 340/380F) | 0.0191 | 0.00346 | 15 | 0.0359 | 0.01166 | 12 | 0.1408 |
| Soma stretch gadolinium  (∆ 340/380F) | 0.0408 | 0.0267 | 69 | 0.00440 | 0.000478 | 127 | 0.0655 |
| Neurite bundle stretch gadolinium  (∆ 340/380F) | 0.0396 | 0.0132 | 37 | 0.00971 | 0.00396 | 12 | 0.2075 |

^n = neurons

Mean responses to soma and neurite bundle stretch, normalized to the response elicited by 60 mM KCl, as well as calcium imaging changes (∆340/380F) under calcium-free and gadolinium conditions. Sample sizes (*n*) indicate the number of neurons analyzed. Statistical comparisons were performed using one-way ANOVA with Tukey’s multiple comparisons test (for stretch responses) and unpaired *t*-tests (for calcium imaging data). Reported *p*-values correspond to specific pairwise comparisons as indicated.

**Table S6. Quantification of iPN and iLTMR responses to increasing probe indentation**

|  | **iPN** | | | **iLTMR** | | |  |
| --- | --- | --- | --- | --- | --- | --- | --- |
|  | **Mean** | **SEM** | **n** | **Mean** | **SEM** | **n** | **Unpaired t-test** |
| I_MA_ at 0.1 µm (pA/pF) | 2.169 | 0.4408 | 26 | 1.85 | 0.2669 | 33 | 0.5199 |
| I_MA_ at 0.2 µm (pA/pF) | 5.312 | 1.426 | 26 | 3.382 | 0.5582 | 33 | 0.1769 |
| I_MA_ at 0.3 µm (pA/pF) | 9.028 | 1.867 | 26 | 5.582 | 0.9993 | 33 | 0.0905 |
| I_MA_ at 0.4 µm (pA/pF) | 13.05 | 2.576 | 26 | 7.501 | 1.318 | 33 | **0.0464** |
| I_MA_ at 0.5 µm (pA/pF) | 15.92 | 3.003 | 26 | 8.946 | 1.449 | 33 | **0.0295** |
| I_MA_ at 0.6 µm (pA/pF) | 18.47 | 3.272 | 26 | 10.47 | 1.578 | 33 | **0.0222** |
| I_MA_ at 0.7 µm (pA/pF) | 20.75 | 3.449 | 26 | 10.81 | 1.582 | 33 | **0.0068** |
| I_MA_ at 0.8 µm (pA/pF) | 21.63 | 3.684 | 26 | 11.45 | 1.584 | 33 | **0.0077** |
| I_MA_ at 0.9 µm (pA/pF) | 23.25 | 3.866 | 25 | 12.04 | 1.62 | 33 | **0.005** |
| I_MA_ at 1.0 µm (pA/pF) | 23.57 | 3.891 | 25 | 12.03 | 1.663 | 32 | **0.0047** |

^n = neurons

Mean mechanically activated current (I_MA_) densities (pA/pF) at varying membrane probe indentation depths (0.1–1.0 µm) recorded from iPN and iLTMR neurons. Values are presented as mean ± SEM, with *n* indicating the number of neurons analyzed. Statistical comparisons between groups were performed using unpaired *t*-tests.

**Table S7. Quantification of iPN and iLTMR responses to repeated probe indentation**

|  | **iPN** | | | | **iLTMR** | | | |
| --- | --- | --- | --- | --- | --- | --- | --- | --- |
|  | **Mean** | **SEM** | **n** | **Unpaired t-test** | **Mean** | **SEM** | **n** | **Unpaired t-test** |
| I_MA_ /I_max#1_ at 2nd repeat | 1.08 | 0.1145 | 15 | 0.4908 | 0.8867 | 0.07254 | 20 | 0.1265 |
| I_MA_ /I_max#1_ at 3rd repeat | 1.032 | 0.1241 | 15 | 0.7954 | 0.7817 | 0.0651 | 20 | **0.0018** |
| I_MA_ /I_max#1_ at 4th repeat | 1.061 | 0.1468 | 15 | 0.68 | 0.7284 | 0.06629 | 20 | **0.0002** |
| I_MA_ /I_max#1_ at 5th repeat | 1.056 | 0.1604 | 15 | 0.7312 | 0.7199 | 0.06402 | 20 | **<0.0001** |
| I_MA_ /I_max#1_ at 6th repeat | 1.067 | 0.1556 | 15 | 0.6703 | 0.618 | 0.07315 | 20 | **<0.0001** |
| I_MA_ /I_max#1_ at 7th repeat | 1.053 | 0.1517 | 15 | 0.7311 | 0.581 | 0.06438 | 20 | **<0.0001** |
| I_MA_ /I_max#1_ at 8th repeat | 1.061 | 0.1548 | 14 | 0.6851 | 0.5386 | 0.07102 | 20 | **<0.0001** |
| I_MA_ /I_max#1_ at 9th repeat | 1.05 | 0.1553 | 14 | 0.7401 | 0.5819 | 0.07092 | 20 | **<0.0001** |
| I_MA_ /I_max#1_ at 10th repeat | 1.08 | 0.16 | 12 | 0.5929 | 0.5549 | 0.08256 | 19 | **<0.0001** |

^n = neurons

Ratios of mechanically activated current (I_MA_) normalized to the first maximum response (Imax#1) across repeated membrane probe indentations (2nd to 10th repeats) in iPN and iLTMR neurons. Values are presented as mean ± SEM, with *n* indicating the number of neurons recorded. Statistical comparisons between repeats were performed using unpaired *t*-tests.

**Table S8. Quantification of iPN and iLTMR responses to probe indentation**

|  | **iPN** | | | **iLTMR** | | |  |
| --- | --- | --- | --- | --- | --- | --- | --- |
|  | **Mean** | **SEM** | **n** | **Mean** | **SEM** | **n** | **Unpaired t-test** |
| Indentation required to elicit an action potential (μm) | 0.84 | 0.11 | 14 | 0.48 | 0.095 | 13 | **0.0218** |
| t current decay (ms) | 0.75 | 0.059 | 10 | 0.64 | 0.045 | 10 | 0.16 |
| I_50_ (µm) | 0.42 | 0.027 | 25 | 0.38 | 0.022 | 33 | 0.221 |
| Slope (µm) | 0.16 | 0.011 | 25 | 0.16 | 0.0088 | 33 | 0.98 |

^n = neurons

Summary of parameters including the membrane probe indentation required to elicit an action potential, current decay time constant (τ), half-maximal indentation (I_50_), and slope of the response curve in iPN and iLTMR neurons. Values are presented as mean ± SEM, with *n* indicating the number of neurons analyzed. Statistical comparisons were made using unpaired *t*-tests.

**Table S9: Quantification of the response of iPN and iLTMR to increasing probe indentation following PIEZO2 KD**

|  | **iPN nontargeting siRNA** | | | **iPN PIEZO2 siRNA** | | |  |
| --- | --- | --- | --- | --- | --- | --- | --- |
|  | **Mean** | **SEM** | **n** | **Mean** | **SEM** | **n** | **Unpaired t-test** |
| I_MA_/I_maxVeh_ at 0.1 µm | 0.1363 | 0.02136 | 16 | 0.1198 | 0.03883 | 16 | 0.7132 |
| I_MA_/I_maxVeh_ at 0.2 µm | 0.1831 | 0.03013 | 16 | 0.1192 | 0.03705 | 16 | 0.1907 |
| I_MA_/I_maxVeh_ at 0.3 µm | 0.2899 | 0.06858 | 16 | 0.1306 | 0.03562 | 16 | **0.048** |
| I_MA_/I_maxVeh_ at 0.4 µm | 0.3675 | 0.0927 | 16 | 0.1617 | 0.05348 | 16 | 0.0641 |
| I_MA_/I_maxVeh_ at 0.5 µm | 0.4296 | 0.1005 | 16 | 0.169 | 0.05218 | 16 | **0.0285** |
| I_MA_/I_maxVeh_ at 0.6 µm | 0.5314 | 0.1102 | 16 | 0.1876 | 0.06076 | 16 | **0.0104** |
| I_MA_/I_maxVeh_ at 0.7 µm | 0.6885 | 0.1639 | 16 | 0.1926 | 0.06323 | 16 | **0.0084** |
| I_MA_/I_maxVeh_ at 0.8 µm | 0.786 | 0.1868 | 16 | 0.1961 | 0.06881 | 16 | **0.0059** |
| I_MA_/I_maxVeh_ at 0.9 µm | 0.9622 | 0.2414 | 16 | 0.2073 | 0.07353 | 16 | **0.0055** |
| I_MA_/I_maxVeh_ at 1.0 µm | 0.9375 | 0.2587 | 16 | 0.2287 | 0.07647 | 16 | **0.0134** |
|  | **iLTMR nontargeting siRNA** | | | **iLTMR PIEZO2 siRNA** | | |  |
|  | Mean | SEM | n | Mean | SEM | n | Unpaired t-test |
| I_MA_/I_maxVeh_ at 0.1 µm | 0.1689 | 0.03351 | 15 | 0.03766 | 0.01448 | 17 | **0.0008** |
| I_MA_/I_maxVeh_ at 0.2 µm | 0.3818 | 0.1999 | 15 | 0.04545 | 0.01941 | 17 | 0.0845 |
| I_MA_/I_maxVeh_ at 0.3 µm | 0.478 | 0.2234 | 15 | 0.05746 | 0.02404 | 17 | 0.0554 |
| I_MA_/I_maxVeh_ at 0.4 µm | 0.6174 | 0.282 | 15 | 0.06231 | 0.03077 | 17 | **0.0457** |
| I_MA_/I_maxVeh_ at 0.5 µm | 0.6944 | 0.2944 | 15 | 0.08192 | 0.05096 | 17 | **0.0374** |
| I_MA_/I_maxVeh_ at 0.6 µm | 0.7621 | 0.3028 | 15 | 0.1035 | 0.0623 | 17 | **0.0313** |
| I_MA_/I_maxVeh_ at 0.7 µm | 0.8571 | 0.3346 | 15 | 0.1288 | 0.09059 | 17 | **0.0342** |
| I_MA_/I_maxVeh_ at 0.8 µm | 0.9259 | 0.3501 | 15 | 0.1422 | 0.09901 | 17 | **0.0303** |
| I_MA_/I_maxVeh_ at 0.9 µm | 0.974 | 0.3496 | 15 | 0.1491 | 0.1001 | 17 | **0.0231** |
| I_MA_/I_maxVeh_ at 1.0 µm | 1 | 0.3578 | 15 | 0.1643 | 0.1082 | 17 | **0.0252** |

^n = neurons

**Table S10 Media and Components**

| **Neural Induction Media** | | |
| --- | --- | --- |
| **Reagent** | **Catalogue number** | **Company** |
| Neurobasal medium | 21103-049 | ThermoFisher |
| DMEM/F12 |  | Made in house |
| N-2 supplement (100x) | 17502-048 | ThermoFisher |
| B-27 supplement without vitamin A (50x) | 12587-010 | ThermoFisher |
| Insulin-transferrin-Selenium-A (100x) | 51300-044 | ThermoFisher |
| 2 mM L-glutamine | 25030149 | Life Technologies |
| 0.3% glucose | G8769 | Sigma |
| **Neuronal Media** | | |
| **Reagent** | **Catalogue number** | **Company** |
| Neurobasal medium | 21103-049 | ThermoFisher |
| N-2 supplement (100x) | 17502-048 | ThermoFisher |
| B-27 supplement without vitamin A (50x) | 12587-010 | ThermoFisher |
| Insulin-transferrin-Selenium-A (100x) | 51300-044 | ThermoFisher |
| L-glutamine (100x) | 25030149 | Life Technologies |
| **BrainPhys media** | | |
| **Reagent** | **Catalogue number** | **Company** |
| BrainPhys™ Neuronal Medium | 5790 | STEMCELL^TM^ Technologies |
| NeuroCult™ SM1 Without Vitamin A | 5731 | STEMCELL^TM^ Technologies |
| N2 Supplement-A | 7152 | STEMCELL^TM^ Technologies |

**Table S11: Primary and secondary antibody details and dilutions for immunocytochemistry**

| **Antibody** | **Species** | **Dilution** | **Catalogue number** | **Company** |
| --- | --- | --- | --- | --- |
| ISLET1 | Rabbit | 1:500 | ab20670 | Abcam |
| BRN3A | Mouse | 1:500 | MAB1585 | Millipore |
| ß- III TUBULIN | Mouse | 1:500 | MAB1637 | Millipore |
| PERIPHERIN | Rabbit | 1:500 | Ab4666 | Abcam |
| NF200 | Mouse | 1:500 | N0142 | Sigma |
| TRKA | Goat | 1:400 | RDSAF175 | R&D Systems |
| TRKB | Mouse | 1:100 | NOVNBP147898 | Novus Biologicals |
| TRKC | Rabbit | 1:250 | 7H3L20 | ThermoFisher |
| NGN2 | Rabbit | 1:250 | PA5-78556 | ThermoFisher |
| RUNX3 | Mouse | 1:250 | ab135248 | Abcam |
| SHOX2 | Mouse | 1:500 | ab55740 | Abcam |
| Donkey-anti-mouse IgG-488 | - | 1:500 | ab150109 | Abcam |
| Donkey-anti-rabbit IgG-555 | - | 1:500 | ab150062 | Abcam |
| Donkey-anti-goat IgG-647 | - | 1:500 | ab150135 | Abcam |
| Donkey-anti-mouse IgG-647 | - | 1:500 | ab150111 | Abcam |
| Donkey-anti-sheep/goat IgG-647 | - | 1:500 | ab150111 | Abcam |

**Table S12: Primary and secondary antibody details for western blotting**

| **Antibody name** | **Host** | **Species reactivity** | **Dilution** | **Catalogue number** | **Company** |
| --- | --- | --- | --- | --- | --- |
| NGN2 | Rabbit | Human, Mouse, Rat | 1:3000 | PA5-78556 | ThermoFisher |
| RUNX3 | Mouse | Human, mouse | 1:3000 | ab135248 | Abcam |
| SHOX2 | Mouse | Human, rat | 1:3000 | ab55740 | Abcam |
| GAPDH | Mouse | Human | 1:20,000 | G8795 | Sigma |
| GAPDH | Rabbit | Human | 1:20,000 | G9545 | Sigma |
| Goat Anti-Mouse IgG H&L (HRP) | - | - | 1:10,000 | ab97023 | Abcam |
| Goat Anti-Rabbit IgG Antibody, (H+L) HRP conjugate | - | - | 1:10,000 | ap307p | Merk Millipore |
